# Large-scale movement of eIF3 domains during translation initiation modulate start codon selection

**DOI:** 10.1093/nar/gkab908

**Published:** 2021-10-14

**Authors:** Jose L Llácer, Tanweer Hussain, Jinsheng Dong, Laura Villamayor, Yuliya Gordiyenko, Alan G Hinnebusch

**Affiliations:** Instituto de Biomedicina de Valencia (IBV-CSIC), Valencia 46010, Spain; Centro para Investigación Biomédica en Red sobre Enfermedades Raras CIBERER-ISCIII, Valencia, Spain; Molecular Reproduction, Development and Genetics (MRDG), Biological Sciences Building, Indian Institute of Science, Bangalore 560012, India; Laboratory of Gene Regulation and Development, Eunice K. Shriver National Institute of Child Health and Human Development, National Institutes of Health, Bethesda, MD 20892, USA; Instituto de Biomedicina de Valencia (IBV-CSIC), Valencia 46010, Spain; MRC Laboratory of Molecular Biology, Cambridge CB2 0QH, UK; Laboratory of Gene Regulation and Development, Eunice K. Shriver National Institute of Child Health and Human Development, National Institutes of Health, Bethesda, MD 20892, USA

## Abstract

The eukaryotic initiation factor 3 (eIF3) complex is involved in every step of translation initiation, but there is limited understanding of its molecular functions. Here, we present a single particle electron cryomicroscopy (cryo-EM) reconstruction of yeast 48S ribosomal preinitiation complex (PIC) in an open conformation conducive to scanning, with core subunit eIF3b bound on the 40S interface near the decoding center in contact with the ternary complex eIF2·GTP·initiator tRNA. eIF3b is relocated together with eIF3i from their solvent interface locations observed in other PIC structures, with eIF3i lacking 40S contacts. Re-processing of micrographs of our previous 48S PIC in a closed state also suggests relocation of the entire eIF3b-3i-3g-3a-Cter module during the course of initiation. Genetic analysis indicates that high fidelity initiation depends on eIF3b interactions at the 40S subunit interface that promote the closed PIC conformation, or facilitate the relocation of eIF3b/eIF3i to the solvent interface, on start codon selection.

## INTRODUCTION

Eukaryotic translation initiation is a complicated process that involves a preinitiation complex (PIC) of the 40S subunit and initiation factors that binds to the 5′ end of mRNA and scans the mRNA leader until a start codon is encountered in the ribosomal P site (1). First, the factors eIF1, eIF1A and eIF3 bind to the 40S, which facilitates the recruitment of methionyl initiator tRNA (Met-tRNA_i_) as a ternary complex (TC) with eIF2-GTP. eIF5, a GTPase activating protein (GAP) for eIF2, may be recruited along with TC or in complex with eIF3. The 43S preinitiation complex (PIC) thus formed is recruited to the capped 5′ end of mRNA by the eIF4 group of factors (2,3). This 48S PIC, in an open conformation with the tRNA_i_ not fully engaged with the P site (P_OUT_ state), then scans the mRNA until a start codon is encountered. GTP bound to eIF2 can be hydrolysed during the scanning process, however, the phosphate (P_i_) product remains bound. Recognition of the start codon leads to a conformational change in the PIC to form a scanning-arrested closed complex, with Met-tRNA_i_ more tightly bound (P_IN_ state), accompanied by the release of eIF1 and attendant dissociation of P_i_ (1,4–5).

Among the eIFs, eIF3 is the largest and contains the greatest number of subunits, and is involved in every step of translation initiation, including TC and mRNA recruitment to the PIC, impairing the association of the 40S and 60S subunits, and modulating the fidelity of start codon selection (reviewed in (6)). In mammals, eIF3 contains 13 subunits (a–j); however, in *Saccharomyces cerevisiae*, it is a much smaller complex with only six subunits (including the non-essential j/Hcr1 subunit). The five essential subunits in *S. cerevisiae*, namely, a/Tif32, b/Prt1, c/Nip1, g/Tif35 and i/Tif34, comprise a conserved core complex found in all organisms, that interacts with the seven other subunits found in mammalian eIF3.

eIF3 adopts an expanded conformation when bound to the ribosomal PIC. The PCI (Proteosome-COP9 signalosome-eIF3) domains of the eIF3a/eIF3c heterodimer occupies a position near the mRNA exit channel on the solvent interface, whereas the distinct module formed by the 9-bladed β-propeller domain and RRM domain of eIF3b, the 7-bladed β-propeller domain of eIF3i and a segment of eIF3g (eIF3b-3i-3g) is positioned near the mRNA entry channel on the solvent interface (7–11). The two modules are connected by the C-terminal (Cter) end of eIF3a. A comparison of the structures of yeast and mammalian PICs harbouring eIF3 reveal that the five core subunits (a, b, c, g and i) occupy the same positions on the solvent interface of the 40S subunit irrespective of the subunit complexity of eIF3. However, in the cryo-EM structures of yeast 48S PIC complexes in an open (hereafter referred to as ‘py48S-open’) or closed state (hereafter referred to as ‘py48S-closed’) (12), we observed density for a portion of the eIF3b-3g-3i module positioned on the 40S subunit interface near helix h44, ribosomal protein uS12 and the TC, where it could contribute to the 40S-60S anti-association activity of eIF3. (The phrase ‘subunit interface’ refers throughout to the 40S surface that joins with the 60S subunit in 80S ribosomal complexes.)

The aforementioned py48S-open and py48S-closed 48S PIC complexes contain eIF1, eIF1A, TC, eIF3 and mRNA and mimic a scanning-competent state and a scanning-arrested state of 48S, respectively. The PCI domains of the eIF3a/eIF3c heterodimer were observed at their canonical positions near the mRNA exit channel on the solvent interface; however, at least a portion (if not all) of the eIF3b-3g-3i module was present in an alternative location at the subunit interface. Thus, compared to previous structures of a yeast 40S-eIF1-eIF1A-eIF3 (8) and mammalian 43S PICs (7,9), a striking conformational change in eIF3 was observed in the py48S-open and py48S-closed complexes (12) in which eIF3 appears to encircle the entire 40S rather than being confined to the solvent interface of the 40S. The positioning of portions of the eIF3b-3g-3i module at the 40S subunit interface in these complexes showed how eIF3 can contact TC and eIF1 in the decoding center while remaining anchored through its other subunits/domains to the solvent side of the 40S. The β-propeller density observed on the subunit interface near h44, uS12 and TC was tentatively assigned to eIF3i (12). Although eIF3b also contains a β-propeller domain, it was ruled out because partial density for the β-propeller of eIF3b could be seen at its well-known location at the 40S solvent interface near h16 in the py48S-open and py48S-closed complexes (12).

The positioning of the β-propeller of eIF3i near h44, uS12 and TC in the py48S-open and py48S-closed complexes was questioned in a subsequent study by Simonetti *et al.* (13). In examining a late-stage mammalian 48S initiation complex (IC), these authors observed a density at the GTPase-binding site on the subunit interface of 40S, which they interpreted as the β-propeller of eIF3i and additional density near h44, h5 and uS12 as the RRM domain of eIF3g (13). Next, they docked an improved mammalian model of eIF3b (obtained from PDB: 5A5U; (10)) into the yeast py48S-closed map and suggested that this β-propeller density near h44, uS12 and TC on the 40S subunit interface belongs to the eIF3b β-propeller and not to eIF3i (13). It was proposed that eIF3i and eIF3b relocate to the subunit interface independently at different stages during the initiation pathway (13). However, the density at the GTPase-binding site assigned by these authors to the eIF3i β-propeller and that near h44 assigned to the RRM of eIF3g were later correctly re-interpreted as ABCE1 protein (6,14–15). Hence the model proposed for independent relocation of eIF3i and eIF3b to the subunit interface of 48S PIC seems unlikely.

It is important to identify the correct β-propeller (eIF3i or eIF3b) bound on the subunit interface near h44 and to elucidate the role of this positioning in eukaryotic translation initiation. In the late stage of initiation observed in the py48S-eIF5N complex from yeast, the 48S PIC contains eIF1A, TC, eIF3, eIF5 and mRNA and mimics a scanning-arrested state of the 48S after recognition of the start codon (9). However, eIF1 is not observed in py48S-eIF5N complex and it has been replaced on the 40S platform by the N-terminal domain of eIF5 (eIF5-NTD), and Met-tRNA_i_ is more fully accommodated in the P site in preparation for 60S subunit joining (9). The PCI domains of the eIF3a/eIF3c heterodimer were observed at their canonical positions near the mRNA exit channel on the solvent interface, and interestingly, in this late-stage initiation complex, the entire eIF3b-3i-3g module is found on the solvent side of the 40S (9) in the location observed previously in early-stage 43S PICs (7,8). This led us to hypothesize that the entire eIF3b-3i-3g module bound initially at the solvent interface in the 43S PIC, relocates to the subunit interface at the onset of mRNA attachment or scanning up to the point of start codon selection, and then relocates back to the solvent side for the final steps of initiation. This second relocation step would be required to replace eIF1 at the P site with the somewhat bulkier eIF5-NTD and for 40S-60S subunit joining (9). Hence, we sought to obtain a map of the 48S PIC in an open conformation with improved density for the β-propeller bound near h14 and h44 on the 40S subunit interface in order to correctly assign it to eIF3b or eIF3i.

Here, we present a structure of the yeast 48S PIC in an open scanning-competent state with density for the eIF3b β-propeller and RRM domain clearly present on the 40S subunit interface. Interaction assays and genetic analyses of substitutions of key eIF3b residues predicted to perturb its contacts at the subunit interface support our assignment of this density to the eIF3b β-propeller. A similar position of the eIF3b-3i-3g-3a-Cter quaternary complex on the 40S subunit interface is also observed in a new map we derived for our previous py48S-closed complex by masked classification with signal subtraction around the β-propeller of eIF3b. The genetic analyses indicate that eIF3b interactions at the subunit interface expected to stabilize this closed complex, and other interactions expected to promote relocation of the eIF3b-3i-3g-3a-Cter module back to the 40S solvent side, are critical for efficient utilization of near-cognate start codons *in vivo*.

## MATERIALS AND METHODS

### Expression and purification of components of the 48S complex

*Kluyveromyces lactis* 40S subunits were prepared as described earlier (16). *S. cerevisiae* eIF2 was expressed in yeast while eIF1, eIF1A, eIF5, eIF4A, eIF4B and the eIF4G1:eIF4E complex were expressed in *Escherichia coli* Rosetta (DE3) cells as recombinant proteins and purified as described (9,17–18). eIF3 was also expressed in yeast as previously described (17) but with modifications to avoid eIF5 contamination. To achieve the latter, we replaced the phosphocellulose column with a Q-sepharose column and eluted it with an extended KCl gradient from 100 mM to 1 M (50 column volumes), obtaining two batches of eIF3, one without eIF5, the other enriched in eIF5. Wild type tRNA_i_ was overexpressed and purified from yeast and aminoacylated as described (17). The mRNA expression construct comprised a T7 promoter followed by the 49-nt unstructured mRNA sequence of 5′-GGG[CU]_3_[UC]_4_UAACUAUAAAAAUC[UC]_2_UUC[U C]_4_GAU-3′ (with start codon underlined), cloned between XhoI and NcoI sites in a pEX-A2 plasmid (Eurofins Genomics). mRNA was purified and capped as in (9).

### Reconstitution of the 48S complex

The 43S PIC was reconstituted first, by incubating 40S with eIF1, eIF1A, TC (consisting of eIF2, GDPCP and Met-tRNA_i_), and eIF3 in 40S:eIF1:eIF1A:TC:eIF3 molar ratios of 1:2.5:2.5:2:2, in 20 mM MES (pH 6.5), 80 mM potassium acetate, 10 mM ammonium acetate, 5–8 mM magnesium acetate, 2 mM dithiothreitol (DTT), 1 μM zinc acetate. Separately, an mRNA-eIF4 complex was prepared, containing eIF4G1, eIF4E, eIF4A, eIF4B and capped mRNA in molar ratios of 1.5:1.5:5:2:2.5 with respect to the 40S ribosome, in 20 mM Hepes (pH 7.4), 100 mM potassium chloride, 5 mM magnesium chloride, 2 mM DTT, 3 mM AMPPNP). Previously, eIF4F ratios were optimized mainly by pull-down interaction assays using the individual eIF4F components only. The volume of the mRNA–eIF4 reaction mixture was 5 times smaller than that for the 43S PIC. Both the 43S mixture and the mRNA–eIF4 mixture were incubated separately for 5 min at room temperature before combining them. After incubation for 2 min at room temperature, the sample (at a 40S concentration of 80 nM) was cooled to 4°C and used immediately to make cryo-EM grids without further purification. Assembled PICs were resolved by sucrose density gradient centrifugation followed by SDS-PAGE analysis.

### Electron microscopy

3 μl of the 48S complex was applied to glow-discharged Quantifoil R2/2 cryo-EM grids covered with continuous carbon (of ∼50 Å thick) at 4°C and 100% ambient humidity. After 30 s incubation, the grids were blotted for 2.5–3 s and vitrified in liquid ethane using a Vitrobot Mk3 (FEI). Automated data acquisition was done using the EPU software (FEI) on a Titan Krios microscope operated at 300 kV under low-dose conditions (30 e^–^/Å^2^) using a defocus range of 1.2–3.2 μm. Images of 1.1 s/exposure and 34 movie frames were recorded on a Falcon III direct electron detector (FEI) at a calibrated magnification of 104,478 (yielding a pixel size of 1.34 Å). More than 3000 micrographs (Supplementary Figure S1A) were recorded from two independent data acquisition sessions. Micrographs that showed noticeable signs of astigmatism or drift were discarded.

### Analysis and structure determination

The movie frames were aligned with MOTIONCORR (19) for whole-image motion correction. Contrast transfer function (CTF) parameters for the micrographs were estimated using Gctf (20). Particles were picked using RELION (21). References for template-based particle picking (22) were obtained from 2D class averages that were calculated from particles picked with EMAN2 (23) from a subset of the micrographs. 2D class averaging (Supplementary Figure S1B), 3D classification and refinements were done using RELION (21). Both movie processing (24) in RELION and particle ‘‘polishing’’ was performed for all selected particles for 3D refinement. Resolutions reported here are based on the gold-standard FSC = 0.143 criterion (25) (Supplementary Figure S1C). All maps were further processed for the modulation transfer function of the detector, and sharpened (26). Local resolution was estimated using ResMap (27) (Supplementary Figure S2).

For the py48S-open-eIF3 dataset (Supplementary Figure S1D), a total of 2610 good images were obtained after discarding bad micrographs with ice contamination, signs of astigmatism or drift and with poor CTF values. This set of images was used for particle picking. An initial reconstruction was made from all selected particles (360 729) after 2D class averaging using the yeast 40S crystal structure (PDB: 4V88), low-pass filtered to 60 Å, as an initial model. Next, a 3D classification into 12 classes with fine angular sampling and local searches was performed to remove abnormal or empty 40S/40S-eIF1-eIF1A particles from the data. Three classes (144 292 particles) showed density for TC. Further, two consecutive rounds of masked 3D classifications with subtraction of the residual signal (201) were then performed, by creating masks around the density attributed to the TC and to the different domains of eIF3 at the subunit interface. In the first ‘focused’ 3D classification using the TC mask we isolated 104 792 particles containing a distinct density for TC. These (104 792) particles were then subjected to the second round of ‘focused’ 3D classification using the eIF3 mask, where only 1 class contained eIF3 in high occupancy (13 038 particles, 4.6 Å). This class (with 13 038 particles) was further divided into two classes: (a) 48S PIC in a partially open form (7288 particles; 5.6 Å), where the head of the 40S adopts an intermediate position between that in the closed and open forms; (b) 48S PIC in the fully open form (5750 particles, 5.2 Å), which is conformationally identical to our previously reported py48S-open complex. The latter class, i.e. 48S PIC in the fully open form is py48S-open-eIF3 PIC. The quality of the density for eIF3 at the subunit interface is better in the latter class than that in the partially open form or one that resulted from the combination of the two classes.

We have also reprocessed our previous dataset of the py48S-closed complex (Supplementary Figure S1D) using image processing tools that were not available at the time we published our previous work with the aim of improving the local resolution of eIF3 at the subunit interface by isolating a larger number of particles containing higher occupancy for eIF3. We started with more than 1 182 000 particles after 2D-classification and after an initial reconstruction done with all selected particles, a first attempt of focused classification with subtraction of the residual signal using a mask around the observed eIF3b β-propeller near the TC did not yield a satisfactory result. Instead, we carried out a conventional 3D-classification in 16 classes and selected 4 classes containing clear density for the TC (total of 365 343 particles). Movie-processing/particle polishing was carried out for these particles. Then we performed three consecutive masked 3D classifications with subtraction of the residual signal. In the first round we used mask around the density attributed to the eIF3b β-propeller near the TC (‘bgi mask’) and divided it into four classes. Only 1 class with 128 915 particles was selected. This class was then subjected to second round of masked 3D classifications with subtraction of the residual signal using mask around the tRNA/alpha subunit of eIF2 (‘tRNA/alpha mask’) into five classes. Three classes with a total of 55 915 particles were selected and these particles were subjected to a third round of masked 3D classifications with subtraction of the residual signal using a mask around all the different domains of eIF3 at the subunit interface (‘eIF3 mask’). In this latter classification we obtained three different classes in which only one of them (with 12 586 particles, 5.8 Å) showed a distinct density for all eIF3 subunits at the subunit interface (Supplementary Figure S2). This class with 12 586 particles and at 5.8 Å resolution is py48S-closed-eIF3 PIC.

### Model building and refinement

Most of the model building involved only rigid-body fitting of known structures into density by using either Chimera (28) or Coot (29). Only for a few regions (specified below), we have modeled *de novo*, and these regions are built as backbone trace only (polyalanines). The previous atomic model of py48S-open (PDB: 3JAQ) was placed into density by rigid-body fitting using Chimera(28). Overall densities for eIF3 at the subunit interface were similar in py48S-open-eIF3 and py48S-closed-eIF3 maps, so model building for eIF3 (Supplementary Figure S3) was done simultaneously in both maps, using one or another depending on the quality of the map for each of the different domains of eIF3. Initially, all the different domains of eIF3 were fitted by rigid-body only using Chimera as follows:

- The eIF3a/eIF3c PCI dimer was taken from the py48S-closed complex (PDB:3JAP) and placed in its corresponding density at the solvent side of the 40S in the py48S-open-eIF3 map.
- The eIF3b β-propeller was taken from the PDB:4NOX and placed into the drum-like density below TC, in almost the same orientation to that found in the PDB:5K1H.
- The ternary complex eIF3i/eIF3g/eIF3b-Cterm originally placed into the above mentioned drum-like density was now placed (together with the helical linker connecting the eIF3b β-propeller with its C-terminal helix in contact with eIF3i) into the low-resolution density in close contact with the eIF3b β-propeller, thereby placing the two beta propellers in a somewhat similar arrangement to that found at the solvent side of the 40S (9). For a better fitting of eIF3i, this low-resolution area of the map was de-noised using LAFTER (30).
- The eIF3b RRM and the linker connecting it with the eIF3b β-propeller were taken from PDB:5K1H, and placed almost identically, with minor changes derived from *H. sapiens* to *S. cerevisiae* sequence replacement (see below).
- The eIF3a c-term helix in contact with eIF3b and the eIF3c helical bundle was not displaced from their original position.

Then, each domain of eIF3 was rigid-body fitted in Coot (29) and further model building was also done in Coot v0.8 for only a few parts of the model, including: eIF3c N-term model building (see below); replacement of each amino acid of eIF3b by its counterpart in *S. cerevisiae*; and eIF3a C-term helix model building (see below).

#### eIF3c N-term model building

**Figure 1.**
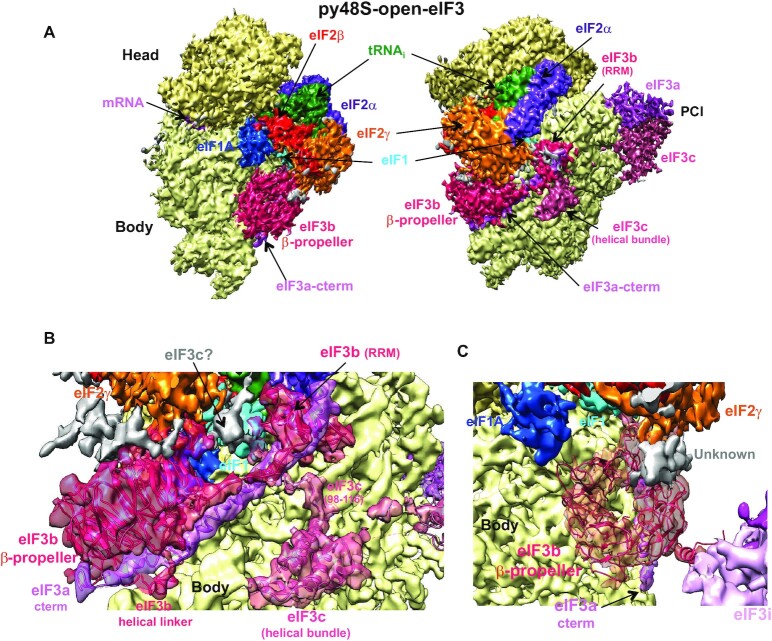
Cryo-EM structure of the py48S-open-eIF3 PIC. (**A**) Cryo-EM maps of the p48S-open-eIF3 PIC shown in two orientations. Regions of the map (at 5.2 Å, threshold of 0.035) are colored by component to show the 40S subunit (yellow), eIF1A (blue), eIF1 (cyan), Met-tRNA_i_^Met^ (green), mRNA (magenta), eIF2α (violet), eIF2γ (orange), eIF2β (red), eIF3 (different shades of pink). The 40S head is shown in a darker yellow compared to the body. The same colors are used in all the figures. Hide dust tool in Chimera at a value of around 50 is also used in all the figures to clean noisy areas from maps. (**B**) Fitting of all of eIF3b, the eIF3a C-term and the eIF3c N-term into the py48S-open-eIF3 map (gaussian-filtered by 1.34 and displayed at threshold of 0.03). (**C**) Fitting of the eIF3b β-propeller into the py48S-open-eIF3 map (gaussian-filtered by 1.34 and displayed at threshold of 0.03).

**Figure 2.**
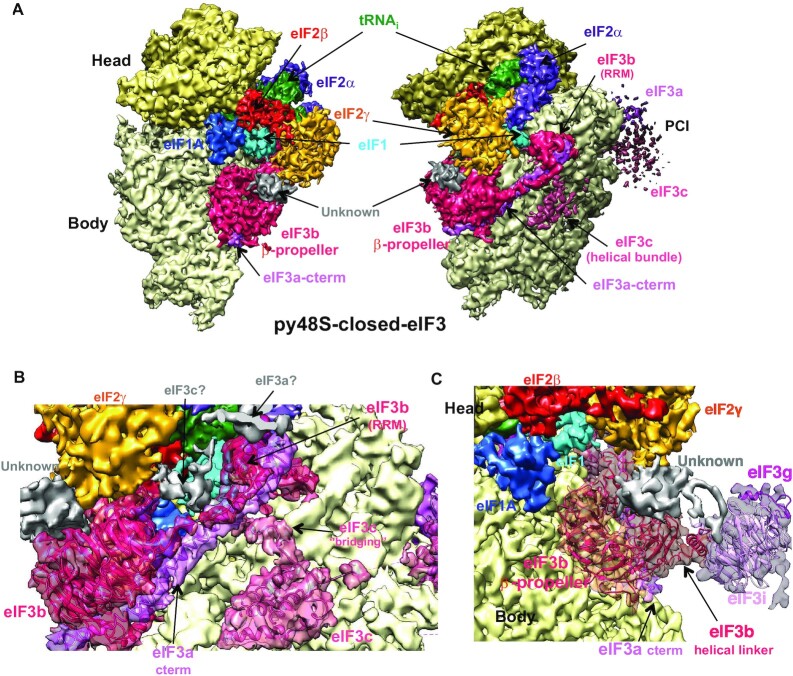
Cryo-EM structure of the py48S-closed-eIF3 PIC. (**A**) Cryo-EM maps (at 5.8 Å, threshold of 0.05) of the py48S-closed-eIF3 PIC shown in two orientations. (**B**) Fitting of eIF3b, the eIF3a C-term and the eIF3c N-term into the py48S-closed-eIF3 map (threshold of 0.04). (**C**) Fitting of the eIF3b and eIF3i β-propellers into the py48S-closed-eIF3 map (threshold of 0.04). Map for eIF3i β-propeller is gaussian-filtered by 1.34 and displayed at threshold of 0.03. An unknown density on top of the eIF3b β-propeller and in contact with eIF2γ is shown in gray.

For the 5-helical bundle model building on the subunit interface near h11/h24/h27 we have used blurred maps of our previous py48S-closed model. Length of the helices matched the lengths predicted by the secondary structure prediction programs and the newer improved maps helped to model the connection between helices (Supplementary Figure S4A–C). Moreover the extra density connecting the helical-bundle with eIF1/RRM and the PCI helped identifying the N- and C-terminus, respectively, of the helical-bundle (residues 117–226). Density for some bulky side chains (Supplementary Figure S4D) also helped in the sequence assignment as well as sequence conservation among homologs from an alignment (Supplementary Figure S4A) of *S. cerevisiae*, *H. sapiens*, *A. thaliana*, *D. rerio*, *D. melanogaster*, and *S. pombe* sequences (we expect that residues of eIF3c involved in interactions are more conserved). In our proposed model for the eIF3c helices, most of the hydrophobic residues are buried and therefore protected from the solvent, whereas charged residues are exposed and may interact with rRNA, eIF1, eIF3b RRM, or other ribosomal proteins (Supplementary Figure S4E, F). Also, a clear eIF3c density (residues 98–116) connecting the eIF3c helical bundle to eIF1 and the eIF3b RRM is observed (Figures 1A, B, 2A, B and Supplementary Figure S4E, F), bridging eIF1 and the eIF3b RRM. This putative ‘bridging’ eIF3c density approaches the residues 50–56 of eIF1 that were earlier proposed to interact with eIF3c (31). Moreover, the interaction in solution of this eIF3c-NTD tail with the eIF3b-RRM and eIF1 was further demonstrated by two different types of *in vitro* interaction assays (see Results section below).

#### eIF3a C-term model building

A long helix of ∼80 residues of the eIF3a C-terminal region is observed running alongside the β-propeller of eIF3b and interacting with both the face of the eIF3b RRM β-sheet and the eIF3b linker connecting the RRM to the β-propeller in both the py48S-open-eIF3 and py48S-closed-eIF3 maps (Figures 1 and 2 and Supplementary Figures S5B and S6D). The density is not clear enough, however, to define unambiguously the side-chains of this eIF3a helix. A completely stretched linker of at least 110 amino acids would be needed to connect the eIF3a helix to the eIF3a PCI domain at the solvent side, a distance of >300 Å around the 40S (Supplementary Figures S3A and S4G and S5C). Based on secondary structure predictions (consensus of programs PSSPred, SOPMA, Jpred), sequence conservation among homologs (alignment done in Clustal Omega using *S. cerevisiae*, *H. sapiens*, *A. thaliana*, *C. elegans*, *D. melanogaster* and *S. pombe* sequences), known biochemical information, and these distance constraints, we have tentatively assigned this linker to the region at the eIF3a C-terminal end spanning residues 693–771. We were also guided by known mutational analysis, i.e. mutations in this region of eIF3a, including those for the KERR motif (32,33) and/or substituting residues 692–701 with Ala (34), are known to impair the binding of eIF3a to the RRM of eIF3b and to the 40S ribosome, respectively. Finally, there is an unassigned density at the extreme C-terminus of the eIF3a helix that also approaches eIF2γ, which probably belongs to eIF3a, but the low resolution of this density and the fact that there are still some segments of factors not accounted for, prevents us from assigning this density unambiguously.

Model refinement was carried out in Refmac v5.8 optimized for electron microscopy (35), using external restraints generated by ProSMART and LIBG (35). Average FSC was monitored during refinement. The final model was validated using MolProbity (36). Cross-validation against overfitting was calculated as previously described (35,37). Buried surface areas and contacts between eIF3b and other 48S components were calculated using PISA (38). All figures were generated using PyMOL (39), Coot or Chimera.

### Plasmid constructions

Plasmids employed in this work are listed in Supplementary Table S3. Plasmids pDH14-29, pDH14-90, pDH15-26, pDH15-67, pDH14-95, pDH14-96, pDH14-56, pDH14-57, pDH15-9, pDH15-10, pDH14-61, pDH15-11, pDH15-76 and pDH15-42 were derived from the low copy number (lc) *LEU2 PRT1* plasmid p5188 by site-directed mutagenesis of *PRT1* using the QuickChange XL kit (Agilent Technologies) and the corresponding primers in Supplementary Table S2. DNA sequence changes were verified by sequencing the entire *PRT1* gene. Plasmids pDH15-59 and pDH15-61 were derived from p5188 and pDH14-90, respectively, by employing the marker swap plasmid pLT11 as described (40), and verified by sequencing. A plasmid for expression of eIF3b RRM domain was kindly donated by Colin E. Aitken (Vassar College, Poughkeepsie, New York) and comprised the region of the *PRT1* gene encoding amino acids 77–162 of eIF3b cloned in a pKYB1 (NEB) vector. Various eIF3b RRM mutant plasmids were obtained by site-directed mutagenesis using the Q5 KIT (NEB) and verified by sequencing the entire *PRT1* gene fragment. A plasmid for GST-eIF3c-NTD1-116 expression was obtained by PCR amplification of a fragment containing the first 348 nucleotides of the *S. cerevisiae NIP1* gene from plasmid pCDFDuet-eIF3a-eIF3c (12), encoding the N-terminal 116 amino acids of yeast eIF3c, and cloned between the SmaI and XhoI sites of plasmid pGEX-6P1.

### Yeast strain construction

Yeast strains employed in this work are listed in Supplementary Table S4. Strain HD3607 was derived from H2995 by replacing the promoter of chromosomal *PRT1* with the *GAL1* promoter by one-step gene replacement, as follows: Primers (i) 5′GAA AAT GCT CCA GTG GCT ACG AAT GCA AAC GCT ACC ACT GAC CAA GAG GGT GAT ATT CAC CTA GAA TAG GAA TTC GAG CTC GTT TAA AC 3′ and (ii) 5′ CTA TGT ACT GAG ATG TTA TTG AAA TAT AAT TCG TAA ATA TTT TTC AAT GTG CGT GGA AGA AAA TTT T CAT TTT GAG ATC CGG GTT TT 3′ were used to amplify by PCR the appropriate DNA fragment from plasmid pFA6a-kanMX6-PGAL1 (p3218), which was used to transform strain HD2955 to KanR, resulting in HD3607. The presence of *PGAL1-PRT1* in HD3607 was verified by demonstrating that the lethality on glucose medium can be complemented by lc *PRT1 LEU2* plasmid p5188 but not by an empty vector, and further verified by colony PCR analysis (online protocol, Thermo Fisher Scientific) of chromosomal DNA with the appropriate primers.

Strains HD3648, HD3687, HD3649, HD3848, HD4059, HD4262, HD4256, HD3850, HD3668, HD3669, HD3861, HD3862, HD3672, HD3955, HD4263 and HD4075 were derived from HD3607 by introducing a *LEU2* plasmid containing the indicated mutated *prt1* allele, or empty vector.

Strains HD4081, HD4082, HD4269. HD3995, HD4257, HD4280, HD4281, HD3749, HD4279, HD4012, HD4268, HD4009 and HD3993 were derived from above *PRT1* or *prt1* mutant strains by introducing the sc *TRP1 SUI5* plasmid p4281, or empty *TRP1* vector YCplac22, as indicated in Supplementary Table S4.

Strain HD4053 was derived from H3956 by replacing the promoter of chromosomal *PRT1* with the *GAL1* promoter by one-step gene replacement, and verified by complementation testing and colony PCR, as described above for strain HD3607. Strains HD4108 and HD4109 were derived from HD4053 by plasmid shuffling to replace the resident lc *URA3 SUI1* plasmid with an lc *LEU2* plasmid containing the *SUI1* or *sui1-K60E* allele. HD4192, HD4193, HD4196 and HD4197 were derived from HD4108 or HD4109 by introducing lc *TRP1* plasmids carrying *PRT1* (pDH15-59) or *prt1-K122R* (pDH15-61), respectively.

### β-Galactosidase assays

Assays of β-galactosidase activity in whole-cell extracts (WCEs) were performed as described previously (41).

### Affinity purification of His8-tagged Prt1 and associated eIF3 subunits.

The eIF3 complex was purified using Ni-NTA-silica resin exactly as described previously (42).

### Expression and purification of GST-eIF3c-NTD1-116 and eIF3b RRM77-162 domains for *in vitro* interaction assays

Plasmids encoding GST-eIF3c-NTD1-116 and eIF3b RRM77-162 (and its variants) were used to transform *E. coli* BL21(DE3) cells. Cells were grown at 37°C in liquid LB medium supplemented with 0.1 mg ml^–1^ of ampicillin until an *A*_600_ of 0.6 was attained, when 0.5 or 0.2 mM isopropyl β-d-thiogalactopyranoside (IPTG) was added for GST-eIF3c-NTD and eIF3b-RRM expression, respectively, and then the cultures were grown for 3h additional hours in the case of GST-eIF3c-NTD cultures or 16 h at 16ºC for eIF3b-RRM cultures, before the cells were harvested by centrifugation. All subsequent purification steps were carried out at 4°C.

Cells expressing GST-eIF3c-NTD were suspended in lysis buffer (20 mM HEPES (pH 7.4), 150 mM KCl, 0.1 mM EDTA, 10% glycerol, 1 mM DTT, 0.1% Triton X-100) supplemented with Protease Inhibitor Cocktail (PIC) (NZYTech) and then broken by sonication and centrifuged for 45’ at 11600 rpm. Supernatant was filtered and loaded into a 5 ml-HiTrap Q HP column (GE Healthcare), pre-equilibrated with 20 mM HEPES (pH 7.4), 100 mM KCl, 10% glycerol and 0.5 mM DTT, mounted on an AKTA fast protein liquid chromatography (FPLC) system, and eluted with a linear gradient of 0.1 to 1.0 M KCl. Fractions containing the protein were pooled and loaded into a GST Trap HP (GE Healthcare) pre-equilibrated with 20 mM HEPES (pH 7.4), 150 mM KCl, 10% glycerol and 0.5 mM DTT. The protein was eluted using the same buffer supplemented with 10 mM reduced glutathione, and the fractions containing the protein were pooled and buffer exchanged into storage buffer by centrifugal ultrafiltration (20 mM HEPES (pH 7.4), 75 mM KCl, 10% glycerol, 2 mM MgCl_2_ and 0.5 mM DTT).

Cells expressing eIF3b RRM and its variants were suspended in intein lysis buffer (20 mM HEPES (pH 7.4), 500 mM KCl, 0.1% Triton X-100 and 1 mM EDTA), supplemented with PIC and then broken by sonication. Proteins were then purified using the IMPACTTM (Intein Mediated Purification with Affinity Chitin-binding Tag) system (NEB) in 5 ml chitin resin gravity packed columns. Columns were washed with 30 ml of intein wash buffer (20 mM HEPES (pH 7.4), 1 M KCl, 0.1% Triton X-100 and 1 mM EDTA), 5 ml of intein cleavage buffer without DTT (20 mM HEPES (pH 8.0), 500 mM KCl, 1 mM EDTA) and 5 ml of intein cleavage buffer with 75 mM DTT. Protein was finally eluted after overnight incubation in 10 ml intein cleavage buffer with 75 mM DTT and then further purified by size exclusion chromatography in a Hi load 16/600 Superdex 75 pre-equilibrated with storage buffer.

All proteins were aliquoted, flash-frozen in liquid nitrogen and stored at -80ºC.

### Pull-down interaction assays

Pull-down assays were carried out using Glutathione agarose resins (ABT) and the Mini Bio-Spin Chromatography columns (BioRad). In each case, 50 μl of resin was pre-equilibrated with 500 μl of storage buffer and then 100 μg of GST-eIF3c-NTD fusion protein was immobilized on the resin and incubated 5 min at room temperature (RT). Afterwards, 25 μg of purified eIF1 and/or eIF3b RRM were added and further incubated 5 min at RT. The resin was washed four consecutive times (with 400, 200, 200 and 100 μl) using storage buffer, and the proteins eluted after a 10 min incubation at RT in 100 μl of elution buffer (storage buffer supplemented with 10 mM reduced glutathione). Equal volumes of the last wash and elution fractions were analysed in a 15% polyacrylamide SDS-PAGE gels and Coomassie blue staining.

The same pull-down protocol was used for experiments comparing the binding strength of GST-eIF3c-NTD to different eIF3b RRM variants, but using a lower amount of protein (50 μg of GST-eIF3c-NTD and 15 μg for each eIF3b RRM mutant). The experiments were done in triplicate.

### Binding assay by biolayer interferometry (BLI)

Biolayer Interferometry binding assays were performed using a BLItz system (Pall ForteBio) and anti-GST sensors (ForteBio). 50 μg/ml of GST-eIF3c-NTD was immobilized on the sensor which previously had been hydrated for 10’ in blitz buffer (20 mM HEPES (pH 7.4), 75 mM KCl, 10% glycerol, 2 mM MgCl_2_, 0.5 mM DTT, 1 mg/ml BSA and 0.005% Tween20). The same buffer was used to prepare the different protein dilutions. At least four different increasing concentrations of each prey protein were utilized. Loading, association and dissociation steps, were measured for 2 min. After each run, the sensors were regenerated using 10 mM glycine (pH 2.0) for 5 s, plus 10 s in 1× PBS, and 5 min in 15% sucrose in 1× PBS; and then rehydrated for 10 min in blitz buffer.

Due to nonspecific signals observed for eIF3b RRM proteins, affinity constants were calculated using in each case the nonspecific binding of the protein at its same specific concentration as a reference. Curve fitting and *K*_d_ calculation were done using BLItz Pro 1.2 software. The experiments were done in triplicate and *K*_d_ constants were calculated with the average of all the data using GraphPad Prism.

### Data resources

Two maps have been deposited in the EMDB with accession codes EMDB: 0057 and EMDB: 0058, for the py48S-open-eIF3 and py48S-closed-eIF3 maps, respectively. Two atomic coordinate models have been deposited in the PDB with accession codes PDB: 6GSM and PDB: 6GSN, for the py48S-open-eIF3 and py48S-closed-eIF3 models, respectively. These models replace previous PDB:3JAQ and PDB:3JAP models (12) and are also linked to previous published maps EMDB: 3050 and EMDB: 3049, respectively (12).

### Supplemental information

Supplemental information includes three movies, four tables and 13 figures.

## RESULTS AND DISCUSSION

### Overall structure of the 48S PIC in an open conformation

The yeast 48S PIC in an open conformation was reconstituted as mentioned in the Material & Methods section. We followed a similar protocol used in our earlier study (12) with the following modifications. An unstructured capped 49-mer mRNA with an AUC near-cognate start codon (5′ GGG[CU]_3_[UC]_4_UAACUAUAAAAAUC[UC]_2_UUC[U C]_4_GAU 3′) and recombinant eIF4 factors (reconstituted eIF4F complex and eIF4B) were used to activate the mRNA in place of the 25-mer uncapped mRNA used previously without activation by eIF4 factors. Further, we added eIF3 purified from yeast rather than recombinant eIF3 used earlier. The reconstitution procedure used here, i.e. with a longer and capped mRNA activated by eIF4 factors and usage of eIF3 purified from yeast is closer to the initiation pathway in yeast cells compared to the earlier method. This sample was used to obtain a reconstruction of a cryo-EM map of a 48S PIC in an open conformation at an overall resolution of 5.2 Å (Figure 1A and Supplementary Figures S1, and S2A, B and Table 1). This PIC, henceforth termed ‘py48S-open-eIF3’, is similar to the py48S-open PIC described previously (12). The resolution of the eIFs is greatest close to the ribosome and declines as one proceeds to the periphery of the complex (Supplementary Figure S2A-B), as observed with other initiation complexes earlier (7–11).

**Table 1.** Refinement and model statistics

	py48S-open-eIF3	py48S-closed-eIF3
**Model composition**		
Non-hydrogen atoms	103 300	103 703
Protein residues	8062	8106
RNA bases	1874	1887
**Refinement**		
Resolution used for refinement (Å)	5.2	5.8
Map sharpening B-factor (Å)	–6	–146
Average B-factor (Å)	–	–
Fourier Shell Correlation (FSC)^a^	0.66	0.73
**Rms deviations**		
Bonds (Å)	0.009	0.007
Angles (°)	1.135	1.092
**Validation (proteins)**		
Molprobity score	3.06	2.69
(Percentile in brackets)	(86th)	(95th)
Clashscore, all atoms	14.19	4.75
(Percentile in brackets)	(87th)	(100th)
Good rotamers (%)	89.3	88.2
**Ramachandran plot**		
Favored (%)	87.1	86.5
Outliers (%)	2.3	3.2
**Validation (RNA)**		
Correct sugar puckers (%)	98.6	97.0
Good backbone conformations (%)	56.4	64.6

^a^FSC = Σ(*N*_shell_ FSC_shell_)/ Σ(*N*_shell_), where FSC_shell_ is the FSC in a given shell, *N*_shell_ is the number of ‘structure factors’ in the shell. FSC_shell_ = Σ(*F*_model_*F*_EM_)/ (√(Σ(|*F*|^2^_model_)) √(Σ(|*F*|^2^_EM_))).

In py48S-open-eIF3 PIC the 40S is in an open conformation and the mRNA channel is widened. The tRNA_i_ is bound to the 40S head and away from the 40S body at the P site. Density for all three subunits of eIF2, i.e. α, β and γ is observed (Figure 1A). eIF1 is bound at the P site in its canonical position identical to that of observed in a 40S-eIF1-eIF1A complex (8). eIF1A occupies the A site; however, its N-terminal tail (eIF1A-NTT) is not observed. Thus, py48S-open-eIF3 PIC is similar to the py48S-open previously described (12); however, we observe better density for eIF3 in the current map, allowing a more complete model of eIF3 in a yeast 48S PIC (Figure 1A–C and Supplementary Figures S3–S5). Whereas density for the eIF3a/eIF3c PCI domains was missing in py48S-open PIC, these domains are visible at the solvent interface (Figure 1A and Supplementary Figure S5A) as well as improved density for the eIF3b-3i-3g-3a-Cter module at the 40S subunit interface (Figure 1A–C and Supplementary Figures S2A, B and S5A, B) in the new py48S-open-eIF3 PIC.

We also observe better density for the five helices of eIF3c (spanning residues 117–216) forming a helical bundle on the subunit interface near h11/h24/h27 (Figure 1B and Supplementary Figures S3A and S4A–F), and our modelling of these eIF3c helices agrees with that obtained from the higher resolution map of a human 48S PIC (43). Clear eIF3c density (residues 98–116) connecting the helical bundle to eIF1 and the eIF3b RRM is also observed (Figure 1B). Additional density attached to eIF1 may correspond to the remaining N-terminal residues (1–96) of eIF3c engaged in additional interactions with eIF1 (Figure 1B, grey density); however, better maps will be required to confirm this last possibility. Density for a long helix of ∼80 residues likely of the eIF3a C-terminal region is also observed at the 40S subunit interface (Figure 1B and Supplementary Figures S3A, S4G and S5B, C). No distinct density for mRNA is seen throughout the mRNA-binding channel of the 40S except for the P site, where AU at positions 1 and 2 of the AUC start codon are observed interacting with the UA of the anticodon of the tRNA_i_ (Supplementary Figure S5D). Overall, the mRNA seems to have minimal interactions with the widened mRNA channel of the 40S subunit in this open conformation of the 48S PIC, consistent with a state conducive to mRNA scanning. No density was observed for eIF4 factors, which may reflect the flexibility of eIF4 factors in the 48S PIC.

In summary, py48S-open-eIF3 represents an open, scanning conformation of the yeast PIC where the eIF3a/eIF3c PCI domains remain bound to the solvent interface but the peripheral eIF3b-3i-3g-3a-Cter module is positioned on the 40S subunit interface, such that eIF3 effectively encircles the 40S subunit.

### Overall structure of the 48S PIC in a closed conformation

We also reprocessed our earlier reported ‘py48S-closed’ PIC (Supplementary Figure S1D) (12) to obtain a map at an overall resolution of 5.8 Å resolution showing clear densities for the different eIF3 domains at the 40S subunit interface, henceforth termed ‘py48S-closed-eIF3’ PIC (Figure 2A–C). The 40S is in a closed conformation and the tRNA_i_ is accommodated at the P site with base-pairing with the AUG codon of the mRNA. eIF1A occupies the A site and the eIF1A-NTT approaches the codon-anticodon duplex. The density for α, β and γ subunits of eIF2 is observed and eIF1 is bound at the P site (Figure 2A). The eIF3 is observed with the PCI domains of the eIF3a/eIF3c heterodimer positioned at the solvent side and the eIF3b-3i-3g-3a-Cter module located at the 40S interface (Figure 2A-C), as observed in the previous py48S-closed and in the new py48S-open-eIF3 PIC described above. Thus, the py48S-closed-eIF3 PIC is similar to the py48S-closed previously described (12), but with better density for eIF3 domains at the subunit interface. The particles used to obtain the new map seem to be relatively more homogenous for the different eIF3 domains at the subunit interface, yielding improved density for these eIF3 domains (Figure 2B, C and Supplementary Figure S2C-D) despite overall lower resolution for the whole PIC compared to py48S-closed.

Overall, py48S-closed-eIF3 represents a scanning-arrested closed conformation of the yeast PIC where the peripheral eIF3b-3i-3g-3a-Cter module is observed at the 40S subunit interface while the eIF3a/eIF3c PCI domains are bound at the solvent interface, just as observed in the py48S-open-eIF3 PIC.

### eIF3b bound on the subunit interface of the 40S

**Figure 3.**
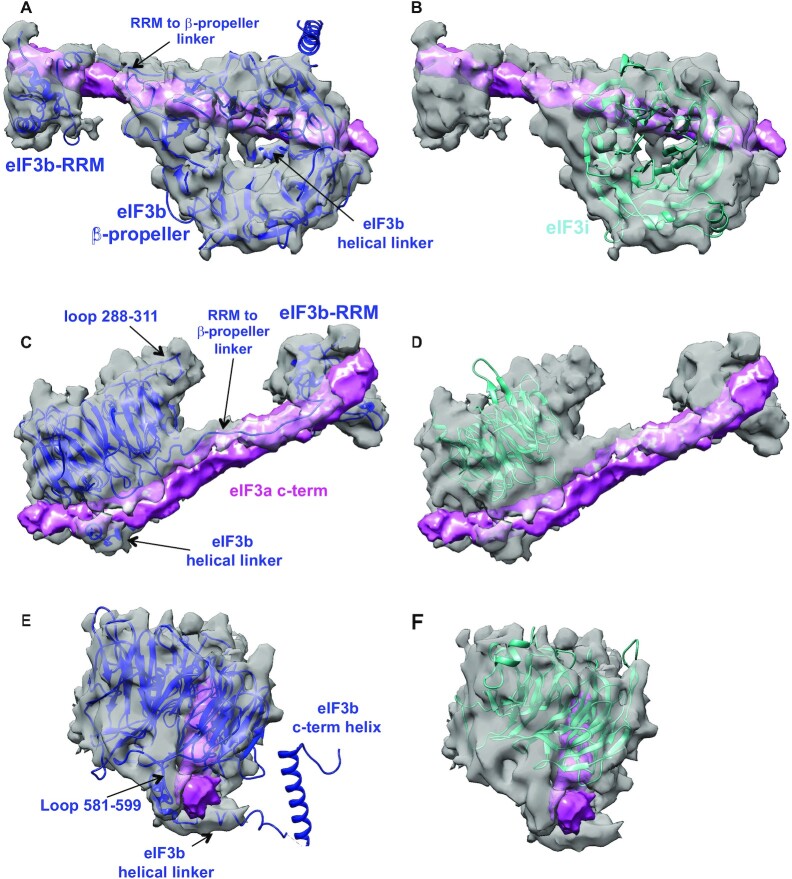
Superior fitting of the structure of the β-propeller of eIF3b versus that of eIF3i. Three different views of the assigned eIF3b density (shown in grey) as observed in py48S-closed-eIF3 map are shown in panels **A–B**, **C–D** and **E–F**, with that assigned to the eIF3a C-term in magenta. The known crystal structure (PDB:4NOX) of the β-propeller of eIF3b, shown in violet ribbon diagram in panels A, C, and E, shows a better fit than that of eIF3i (from PDB: 4U1E), shown in cyan in panels B, D and F, to the eIF3b density. The diameters of the inner and outer rims of the ring-shaped density are more appropriate for those of 9-bladed β-propeller of eIF3b versus the 7-bladed β-propeller of eIF3i. Density corresponding to loops present in eIF3b and not present in the eIF3i β-propeller are also observed and labeled in C and E, as well as for the two linkers connecting the eIF3b β-propeller with eIF3b-RRM (labeled in A and C) or with the eIF3b C-terminal helix (labeled ‘eIF3b helical linker’ in A, C and E).

Density for a β-propeller on the 40S subunit interface near h14 and h44 and in contact with eIF2γ was observed in both py48S-open-eIF3 and py48S-closed-eIF3 maps (Figures 1 and 2). We assign this density to the β-propeller of eIF3b, as it shows a decidedly better fit to the density compared to that of eIF3i (Figure 3A-F and Supplementary Figure S6A-B). First, the diameter of the inner and outer rims of the ring-shaped density is more appropriate for the size of the 9-bladed β-propeller of eIF3b than that of the 7-bladed β-propeller of eIF3i (Figure 3A–F). Second, density corresponding to loops present in eIF3b and not in the eIF3i β-propeller are also observed (Supplementary Figure S6A, B). Thus, we agree with Simonetti *et al.* (13) that the β-propeller of eIF3b instead of the β-propeller of eIF3i is bound near h14 and h44 in the previous py48S-closed map, and we observe this binding here as well in the py48S-open-eIF3 and py48S-closed-eIF3 maps. Also, in these maps we do not observe any clear density for the β-propeller of eIF3b at the solvent interface near h16. It is likely that our previously reported py48S-open and py48S-closed maps (12) had a small fraction of 48S PIC particles with the eIF3b β-propeller in its canonical position at the solvent interface (7–11) in addition to those harbouring this eIF3b domain at the subunit interface.

The eIF3b β-propeller is anchored on the 40S subunit interface between h14, h15, h44 and ribosomal protein uS12 (Supplementary Figure S6C-D). Although the β-propeller is positioned in proximity of eIF1A, no direct interaction between the two was observed (Figures 1C and 2C). However, the eIF3b β-propeller is in contact with domain III of eIF2γ (Figures 1A-C and 2A-C and Supplementary Figures S6B-D), and also with the long helix in the eIF3a C-terminal region mentioned above (Figures 1B, 2B and 3C). Density for the eIF3b RRM domain is also observed in both py48S-open-eIF3 and py48S-closed-eIF3 PICs (Figures 1B, 2B, 3A and B) interacting with h24 at the 40S platform (Supplementary Figure S6D). The eIF3b RRM also interacts with the long helix in the eIF3a C-terminal region (Figures 1B, 2B, 3A and B), and with the eIF3c N-terminal tail (eIF3c-NTD tail) and eIF1 (Supplementary Figure S6D). These contacts of eIF3b RRM with eIF1 and eIF3c-NTD tail seem to be unique to both py48S-open-eIF3 and py48S-closed-eIF3 PICs, as the RRM is distant from both eIF1 and the eIF3c-NTD tail in previous py43S structures (7–8,10–11) and in the late-stage py48S-eIF5N PIC (7,9,11).

**Figure 4.**
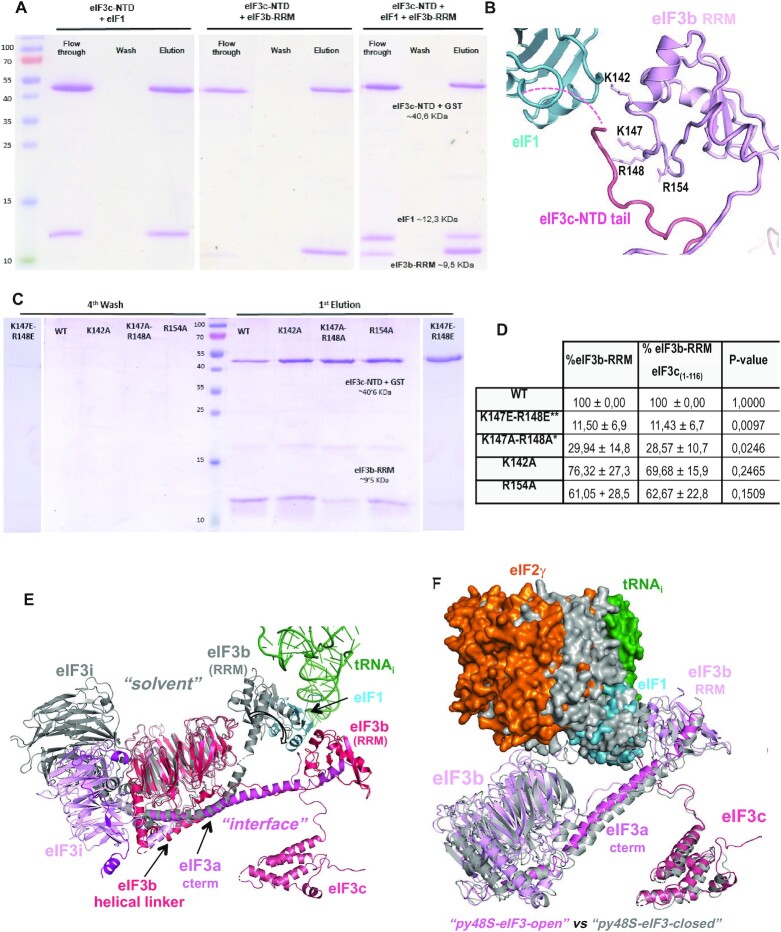
eIF3b undergoes internal rearrangements and its eIF3b RRM domain interacts with eIF3c-NTD and eIF1. (**A–D**) Biochemical evidence supporting eIF3b relocation to the 40S subunit interface from GST pull-down assays involving eIF3c-NTD, eIF3 RRM and eIF1, predicted to form a ternary complex only in the py48S-open-eIF3 and py48S-closed-eIF3 complexes. (A) Recombinant GST-eIF3c-NTD_1-116_ immobilized on glutathione agarose resin was incubated with purified eIF1, eIF3c-NTD, or both, washed extensively, and eluted with glutathione. Equal proportions of flow-through, the last wash, and eluate fractions were resolved by SDS-PAGE and stained with Coomassie Blue. (B) Cartoon representation showing the area of contact between eIF1 (cyan), eIF3b RRM (light pink) and eIF3c-NTD tail (dark pink). Residues selected for mutagenesis and used for in vitro interaction assays are indicated. (C) Pull down assays of interactions between GST-eIF3c-NTD_1-116_ and different eIF3b RRM mutants, performed as in (A). (D) Table summarizing densitometry of the gel bands shown in (C) for the eIF3b RRM bands alone (column 2) or the eIF3b RRM bands normalized to the corresponding intensities of GST-eIF3c-NTD in the same lane. Asterisks indicate significant differences between mutant and WT as judged by a Student's *t*-test (*P < 0.05, **P < 0.01). (**E**) Superimposition of the eIF3b/eIF3i/eIF3g/eIF3a-Cterm quaternary complex observed in py48S-closed-eIF3 with that found on the 40S solvent side in py48S-eIF5N (in grey; PDB: 6FYY), aligning the eIF3b β-propellers, shows how this eIF3 subcomplex undergoes internal rearrangements in the transition between the two states, possibly resulting from constraints imposed by its interactions with eIF2 and eIF1 unique to the subunit interface location. (**F**) Superimposition of eIF3 subunits in the py48S-closed-eIF3 and py48S-open-eIF3 structures, achieved by aligning the 40S bodies in both structures. For clarity, the eIF3i/eiF3g subunits are not shown. tRNA_i_, eIF2 and eIF1 are shown as surfaces. Other components of the py48S-closed-eIF3 structure are colored gray.

To validate these interactions of the eIF3b RRM, we carried out *in vitro* interaction assays using purified eIF1, eIF3b RRM and GST-eIF3c-NTD (spanning residues 1 to 116), which demonstrated that the eIF3c-NTD interact directly with both eIF1 and the eIF3b RRM in solution (Figure 4A). While the interaction of eIF1 with eIF3c-NTD is consistent with previous results, the eIF3c–NTD interaction with eIF3b RRM has not been described previously (6). Importantly, substitutions of key basic residues of the eIF3b RRM, Lys-147 or Arg-148, predicted to perturb its interaction with the eIF3c-NTD (Table 2), reduced the amount of eIF3b RRM recovered with GST-eIF3c-NTD (Figure 4B-C) and substantially decreased the binding strength between the two proteins determined by biolayer interferometry (BLI) experiments (Supplementary Figure S7). In contrast, substitutions of two nearby eIF3b RRM basic residues not predicted to contact eIF3c-NTD, Lys-142 and Arg-154, produced relatively smaller reductions in the pull-down assays (Figure 4B–D) and did not alter the binding strength measured by BLI (Supplementary Figure S7).

**Table 2.** Possible interactions of eIF3b residues with the 40S subunit or other eIFs in the py48S-open-eIF3 or py48S-closed-eIF3 complexes^a^

eIF3b residue	Interacting residue(s) in py48S-open-eIF3	Interacting residue(s) in py48S-closed-eIF3	Interacting residue in py48S-eIF5N complex (9)
P88	rRNA 986G	same	None
K91	rRNA 1011U	rRNA 1011U, 1012A and 986G	None
S101	rRNA 995U	rRNA 994A	None
K105	R53 and D50 of eIF1, D97 and phosphoSer98 of eIF3c	None	None
T120	None	rRNA 984G, 985G	None
K122^d^*Stabilization of open 48S PIC*	rRNA 984G and 985G	rRNA 985G	None
K142	D50 and Y49 of eIF1, NTD of eIF3c	None	None
S143	R53 of eIF1,Y96 of eIF3c	None	None
K147^d^*Stabilization of closed 48S PIC*	Y96 and phosphoSer99 of eIF3c	same	None
R148	S98, phosphoSer99, D100 and phosphoSer103 of eIF3c	phosphoSer99, D100 and phosphoSer103 of eIF3c	None
D150	rRNA 1011U	same	None
K152^d^*Stabilization of closed 48S PIC*	rRNA 982A and 1012A	rRNA 981U and 982A	None
Q250	K252 of eIF2γ	R454 and K252 of eIF2γ	None
Y268	R454 of eIF2γ	same	None
D271	None	K252 of eIF2γ	None
E290	R439 of eIF2γ	unknown density on top of eIF3b propeller (eIF3g?, eIF5 CTD?)	None
I293	K452 of eIF2γ	I505 and E506 of eIF2γ	None
V294	A451 of eIF2γ	I503 of eIF2γ	None
E295	R454 of eIF2γ	R454 of eIF2γ	None
E296	None	R504 and K207 of eIF2γ	None
D297^d^*facilitates relocation of the eIF3b-3i-3g-3a-Cter module to the 40S solvent exposed surface*	D457 and R454 of eIF2γ	D460 of eIF2γ	None
E308^d^*facilitates the relocation of the eIF3b-3i-3g-3a-Cter module to the 40S solvent exposed surface*	Residues around 445–448 of eIF2γ	E506 and possibly also K449 of eIF2γ	None
K346^d^*Stabilization of open 48S PIC*	rRNA 1742A	None	None
K363	rRNA 1743G and 1744A	rRNA 1745G	None
M366	rRNA 1745G	None	None
F393	Q75 of uS12	None	None
R394	rRNA 433G and I77 of uS12	Q75 and L76 of uS12	rRNA 780A
N395	rRNA 46A	rRNA 433G	None
G396	None	rRNA 1742A	None
D397^d^*facilitates the relocation of the eIF3b-3i-3g-3a-Cter module to the 40S solvent exposed surface*	None	rRNA 1743G	None
E398^d^*facilitates the relocation of the eIF3b-3i-3g-3a-Cter module to the 40S solvent exposed surface*	None	rRNA 1743G	None
R424	Q75 of uS12	L54 and E55 of uS12	None
R426	E 98 and N99 of uS12	N99 of uS12	None
V427	None	N99 of uS12	None
R476	rRNA 431G	E101 of uS12	rRNA 778G
D477	None	S145 of us12	H155 of uS4.
E530	rRNA 413C	None	None
K531	rRNA 413C and 415A	rRNA 413C	rRNA 676G
T532	rRNA 412U and 413C	None	None

^a^At the current resolution, side chains of residues are not well resolved, and therefore this table reflects possible interactions with residues in close proximity to a given eIF3b residue. As a consequence, in some cases we include more than one possible interacting residue. Possible contacts of eIF3b residues located in the helical linker between the β-propeller and the C-terminal-eIF3i-interacting helix (residues 668–701) with the 40S and eIF3a-cterm are not mentioned here because we have modeled ‘*de novo*’ only the backbone but not the side chains for residues on this region.

^b^Interactions of eIF3b with eIF3a CTD are not mentioned as they are largely similar in the OPEN and CLOSED states and may involve residues Y78, V80, N82, E113, F126, F128, Y158, V163, Y166, N167, N170, D172, T173, F175, E177, P178, P181, T182, P185, S187, K190, L193, M194, V198, R559, F560, D580, Y583, P584, G585, K600, V602, R620, I642, A643, R663, residues of the helical linker (see above).

^c^Interactions of eIF3b with 3i are not mentioned because of the poor local resolution of this part of the map. In any case, we do not expect differences in the contacts between eIF3i and eIF3b to those observed in the high resolution crystal structures of eIF3i-eIF3b yeast complexes, PDB: 3ZWL or PDB: 4U1E.

^d^Residues mutated in genetic studies and role of that residue on translation initiation inferred from genetic studies.

All of the possible interactions of eIF3b residues with rRNA, 40S proteins, or other eIFs at the subunit interface in the open and closed conformations are listed in Table 2. Inspection of these data indicates that interactions of the eIF3b β-propeller and RRM with the 40S or the initiation factors eIF1 and eIF2 promote a distinctly different conformation of the eIF3b/eIF3a–CTD subcomplex at the subunit interface compared to the late-stage py48S-eIF5N where the same subcomplex is bound at the 40S solvent interface (Figure 4E). In addition, we observe a subtle repositioning of the β-propeller in the closed state compared to the open state that leads to some differences in its interactions with the 40S or eIF residues between the two states (Figure 4F and Movie 1). Some residues of eIF3b are predicted to make the same interactions with the 40S or eIF residues in both states, whereas a few residues likely engage in a certain interaction only in the open or closed complex (Table 2; see also genetic studies below), including direct contact between the eIF3b RRM and eIF1 observed exclusively in the open state (Table 2). The latter interactions should help keep eIF1 positioned at the P site during scanning, while the lack of eIF3b/eIF1 interaction in the closed state should favour eIF1 release from the PIC after recognition of the start codon. Interestingly, in the closed state only, several acidic side chains of the eIF3b β-propeller (Glu308, Asp397, Glu398) seem to be positioned in proximity to the negatively charged phosphate residues of rRNA or acidic side chains of eIF2γ (Table 2), consistent with electrostatic repulsion. This repulsion might help to promote the re-positioning of eIF3b back to the solvent interface after eIF1 leaves the PIC. Similar repulsion was predicted between eIF1 and Met-tRNA_i_ in the closed-state PICs where acidic side chains of eIF1 are positioned in proximity to the negatively charged phosphate residues of tRNA_i_ (44) and shown to promote initiation accuracy *in vivo* (45); and similarly between IF3 and fMet-tRNA_i_ in the closed state of bacterial PICs (46).

In summary, the β-propeller of eIF3b binds on the 40S subunit interface near h14 and h44, making direct contacts with rRNA and ribosomal proteins in both the py48S-open-eIF3 and py48S-closed-eIF3 PICs. In both complexes, the eIF3b β-propeller also makes contacts with eIF2γ while the eIF3b RRM domain interacts with the eIF3a C-terminal helical region, the eIF3c-NTD tail and eIF1.

### Trimeric complex of eIF3i-3g-3b-C-terminal helix bound to the eIF3b β-propeller also relocates to the subunit interface of the 40S

In its canonical location in the 43S PIC, the C-terminal helix of eIF3b interacts with the β-propeller of eIF3i at the solvent side of the 40S, where eIF3i makes no direct contact with the 40S (6,10,47). In our new maps the density for almost the entire eIF3b, excluding the C-terminal helix, can be accounted for at the subunit interface (Figures 1B, 2C and Supplementary Figure S3B). Interestingly, using low-pass or gaussian filtered maps, density for the trimeric complex eIF3i-eIF3g-eIF3b C-terminal helix can be observed in proximity to the eIF3b β-propeller in both py48S-open-eIF3 and py48S-closed-eIF3 PICs (Figures 1C and 2C, Supplementary Figures S8 and S9). Density for the trimeric complex eIF3i-eIF3g-eIF3b C-terminal helix is more resolved in the filtered map of py48S-closed-eIF3 PIC compared to that of py48S-open-eIF3 PIC (Supplementary Figures S8 and S9). Thus, it appears that both eIF3b and eIF3i and the regions of eIF3g interacting with eIF3i move together as a module to the subunit interface from the solvent surface (Supplementary Figure S5C) by virtue of an extended conformation of eIF3a-Cter (Movie 2). The β-propeller of eIF3i appears to be flexible and not restricted to one conformation, resulting in weak density, and it does not contact the 40S. Thus, in the new py48S-open-eIF3 and py48S-closed-eIF3 maps, the eIF3b β-propeller and its RRM domain are present on the 40S subunit interface and eIF3i relocates together with eIF3b to the subunit interface (Supplementary Figure S10).

There is an unassigned density on top of the β-propeller of eIF3b in contact with eIF2γ (Figures 1C, 2A–C), which is more prominently seen in py48S-closed-eIF3 (Figure 2A-C, Supplementary Figures S6B, S7 and S8), which might correspond either to the RRM domain of eIF3g based on its size and proximity to eIF3i or to the C-terminal domain of eIF5 based on the known contacts of eIF2γ with the structurally homologous HEAT domain of eIF2Bϵ (48,49). Further studies are required to identify it with confidence.

In summary, the peripheral eIF3b-3i-3g-3a-Cter module relocates as a unit to the 40S subunit interface and is observed in both the py48S-open-eIF3 and py48S-closed-eIF3 PICs.

### eIF3b interactions at the interface of the 48S PIC modulate the fidelity of start codon selection *in vivo*

The RRM domain of eIF3b/Prt1 contacts eIF1 and the eIF3c-NTD tail, and the β-propeller of eIF3b contacts eIF2γ, at the 40S subunit interface of both the py48S-open-eIF3 and py48S-closed-eIF3 complexes (Figures 1B-C and 2B–C, and Supplementary Figures S5B, S6C-D and S11A-B). It is possible that these interactions stabilize the open conformation and promote scanning, e.g., by helping to anchor eIF1 on the 40S platform. Alternatively, certain interactions might be more important for stabilizing the closed conformation from which eIF1 will be released on start codon recognition, followed by the relocation of the eIF3b-3i-3g-3a-Cter module from the subunit interface back to the solvent-exposed surface of the 40S subunit. Presumably, this relocation is necessary to allow eIF5-NTD to occupy the position of eIF1, dissociation of eIF2 from Met-tRNA_i_ and subsequent joining of the 60S subunit (9). We reasoned that if particular eIF3b contacts at the subunit interface are relatively more important for stabilizing the open, scanning conformation, then substitutions perturbing these contacts would shift the system to the closed state and allow inappropriate selection of near-cognate UUG codons *in vivo*, conferring the ‘Sui^–^’ phenotype. If instead the contacts preferentially stabilize the closed conformation, or impede the transition from open to closed states, then disrupting them should shift the equilibrium to the open scanning conformation and suppress initiation at UUG codons, for the ‘Ssu^–^’ phenotype (reviewed in (1,4)). The Ssu^–^ phenotype might also be conferred by substitutions that impede relocation of the eIF3b-3i-3g-3a-Cter module from the subunit interface of the closed state back to the solvent side of the 40S, which could result either from strengthening eIF3b contacts on the interface surface or by weakening its contacts on the solvent surface.

To examine the phenotypes of eIF3b substitutions *in vivo*, we generated the appropriate mutations in a *PRT1* gene tagged at the C-terminus with the His8 epitope on a single-copy plasmid, and introduced the resulting mutant or WT plasmids into a yeast strain in which expression of the chromosomal *PRT1* allele is under control of the galactose-inducible, glucose-repressible *GAL1* promoter. The resulting transformants, isolated on galactose medium, were tested for mutant phenotypes on glucose medium in which expression of the WT chromosomal *PRT1* allele is transcriptionally repressed. The elevated utilization of UUG start codons in Sui^–^ mutants was identified by testing for suppression of the inability of a his4-303 strain to grow on medium lacking histidine, as his4-303 eliminates the AUG start codon, and Sui^–^ mutations enable initiation at the third in-frame UUG codon of HIS4. Assaying matched HIS4-lacZ reporters with a UUG or AUG start codon yields the UUG:AUG initiation ratio, which is elevated by Sui^–^ mutations. Ssu^–^ phenotypes are scored by testing for suppression of both the His^+^ phenotype and elevated *HIS4-lac*Z UUG:AUG initiation ratio conferred in a *his4-303* strain by *SUI5*, a dominant Sui^–^ allele of *TIF5* encoding eIF5-G31R (50). Ssu^–^ mutations also generally suppress the inability of *SUI5* strains to grow at 37°C (51), which we also scored in all of the mutants.

(i) Evidence that possible interactions of eIF3b RRM residues Lys-147 and Lys-152 with eIF3c-NTD and rRNA on the 40S interface preferentially stabilize the closed conformation of the 48S PIC.

**Figure 5.**
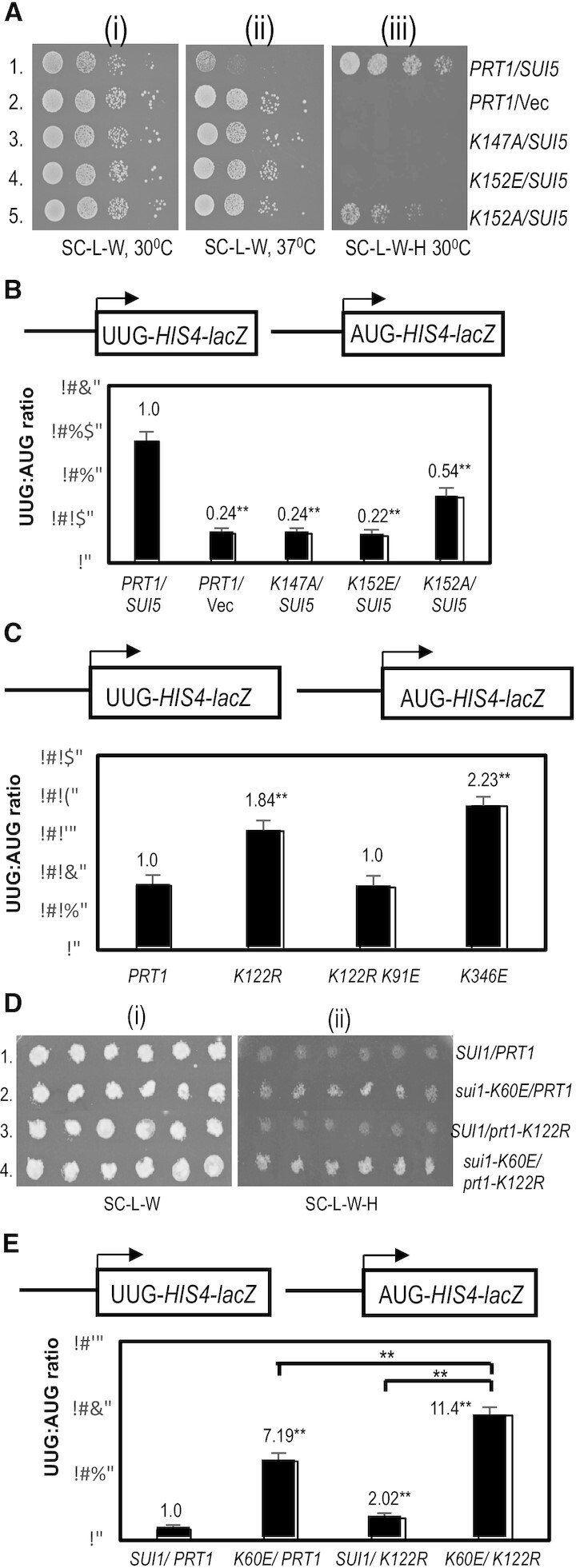
Effect of substitutions in the eIF3b RRM on the fidelity of start codon selection in vivo (A)-(B) Genetic evidence that weakening interactions of eIF3b RRM residues Lys147 and Lys152 at the 40S subunit interface preferentially destabilize the closed conformation of the 48S PIC and increases discrimination against start UUG codons. (**A**) Serial dilutions of transformants of *P_GAL1_-PRT1 his4-301* strain HD3607 with the indicated *PRT1* alleles on low-copy (lc) *LEU2* plasmids and either single-copy (sc) *TRP1 SUI5* plasmid p4281 or empty vector YCplac22 (Vec) were spotted on synthetic complete medium lacking Leu and Trp (SC-L-W) or the same medium also lacking His (SC-L-W-H) and incubated at 30ºC or 37ºC for 3–5d. (**B**) The same strains as in (A) harboring *HIS4-lacZ* fusions with AUG or UUG start codons (shown schematically) on plasmids p367 and p391, respectively, were cultured in synthetic minimal medium containing His (SD + His) to an OD_600_ of 1.0–1.2 and β-galactosidase activities were measured in whole-cell extracts. Ratio of mean expression of the UUG and AUG reporters from six transformants are plotted with error bars indicating SEMs. Asterisks indicate significant differences between mutant and WT as judged by a Student's t-test (*P < 0.05, **P < 0.01). (**C–E**) Evidence that weakening interactions of RRM residue Lys122 at the 40S subunit interface preferentially destabilizes the open conformation of the PIC and elevates initiation at UUG start codons (C) Transformants of strain HD3607 with the indicated *PRT1* alleles and *HIS4-lacZ* fusions with AUG or UUG start codons were cultured in SD + His + Trp, and analyzed exactly as in (B). (D) Transformants of *his4-301* strains HD4108 (*SUI1*) or HD4109 (*sui1-K60E*) with the indicated *PRT1* alleles on lc *TRP1* plasmids were replica-plated to SC-L-W or SC-L-W-H and incubated at 30ºC for 3–6 days. (E) *HIS4-lacZ* UUG:AUG ratios were determined for strains in (D) exactly as in (B).

Lysine-147 in the eIF3b RRM appears to contact the eIF3c-NTD (Figure 4B-D and Supplementary Figures S7 and S12) in the vicinity of Tyr-96 and phosphorylated Ser-99 in both py48S-open-eIF3 and py48S-closed-eIF3 (Table 2). Substituting this Prt1 residue with alanine (K147A) confers a strong Ssu^–^ phenotype, suppressing the Slg^–^ at 37°C, His^+^ phenotype at 30°C, and the elevated UUG:AUG *HIS4-lacZ* initiation ratio conferred by *SUI5* (Figure 5A, row 3 versus row 1, and Figure 5B, colums 1 and 3) (See Supplementary Table S1 for a summary of phenotypes of all *prt1* alleles described in this study). The same three phenotypes characteristic of strong Ssu^–^ mutations were additionally observed for Glu substitution of Lys-152 (K152E) (Figure 5A, rows 1 and 4; Figure 5B, columns 1 and 4), which is also in the RRM and appears to contact rRNA helix 24 (Supplementary Figure S11), including residues A982 and A1012 in the open complex and U981/A982 in the closed complex (Table 2). Interestingly, substituting K152 with Ala strongly suppressed the Slg^–^, but only partially reversed the His^+^ phenotype of *SUI5*, and produced a smaller reduction in the UUG:AUG initiation ratio in *SUI5* cells compared to the K152E substitution (Figure 5A, rows 1 and 5; Figure 5B, cols. 1 and 5 versus 4). The weaker Ssu^–^ phenotype of K152A compared to K152E is consistent with the idea that the Glu substitution introduces electrostatic repulsion with the rRNA phosphate backbone, whereas the Ala substitution would merely eliminate the electrostatic attraction between the Lys side-chain of K147 and the rRNA backbone. None of these substitutions affects cell growth in strains lacking *SUI5* (Supplementary Figure S12A, rows 6–8 versus 1), which is typical of Ssu^–^ substitutions in eIF1 (52).

The residues K147 and K152 appear to make contacts at the subunit interface in both py48S-open-eIF3 and py48S-closed-eIF3. Hence, it was possible that substituting these residues would confer dual Sui^–^ and Ssu^–^ phenotypes, as has been observed previously for certain substitutions in eIF5 (53). At odds with this possibility, however, neither K152E (Supplementary Figure S12B), K152A, nor K147A (Supplementary Figure S12E) increase the UUG:AUG initiation ratio in otherwise WT cells lacking *SUI5*. These findings suggest that the contacts made by K147 and K152 at the subunit interface are not critically required to stabilize the open, scanning conformation of the PIC and, rather, exclusively stabilize the closed state. The selective effect of K152E and K152A on the closed complex might involve the fact mentioned above that K152 may make a different pair of rRNA contacts in the open and closed states. For K147, which appears to make the same interactions in both states, it could be proposed that loss of its eIF3c interaction in the open complex conferred by K147A is compensated by one or more other interactions in the PIC that uniquely stabilize the open complex.

(ii) Evidence that possible interactions of eIF3b RRM residue Lys-122 and β-propeller residue Lys-346 with rRNA on the 40S interface preferentially stabilize the open conformation of the 48S PIC.

eIF3b residue Lys-122 in the RRM appears to contact rRNA helix 24 (Supplementary Figure S11), including residues G985–G986 in the open complex, but only G985 in the closed complex (Table 2). Substituting Lys-122 with Ala, Asp, or Glu, designed to eliminate contact with the rRNA (K122A) or replace it with electrostatic repulsion (K122D and K122E), all conferred dominant lethal phenotypes. This was inferred from our inability to recover transformants containing the corresponding *prt1* mutant alleles even on galactose medium where WT eIF3b/Prt1 is expressed from the chromosomal *PGAL-PRT1* allele. Interestingly, whereas Arg substitution of this same residue (K122R) has no effect on cell growth, it produces a weak Sui^–^ phenotype, increasing the UUG:AUG *HIS4-lacZ* reporter initiation ratio by ≈1.8-fold (Figure 5C, columns 1–2). One way to explain these findings is to propose that interaction of K122 with rRNA residues on the 40S interface is essential, such that its replacement with Ala, Asp, or Glu is lethal. The dominance of this lethal phenotype indicates that the mutant protein is expressed and competes with WT eIF3b/Prt1 for incorporation into the eIF3 complex. The basic side-chain introduced by K122R would maintain the essential electrostatic attraction with rRNA, but the larger size of the Arg versus Lys side-chain would perturb RRM binding to the rRNA in a manner that diminishes its ability to stabilize the open complex. Consistent with this interpretation, we observed that K122R exacerbates the His^+^/Sui^–^ phenotype and elevated *HIS4-lacZ* UUG:AUG ratio conferred by the sui1-K60E substitution, which is known to weaken eIF1 binding to the 40S subunit, destabilizing the open complex and increasing inappropriate transition to the closed state at UUG codons (54) (Figure 5D, rows 2 and 4 versus 1; and Figure 5E, columns 2 and 4 versus 1).

We found that the K122R substitution does not confer an Ssu^–^ phenotype in the presence of *SUI5* (Supplementary Figure S12C), suggesting that it either does not appreciably destabilize the closed complex, or that it confers a relatively greater impairment of the open complex with the net effect of only a weak Sui^–^ phenotype. The relatively greater destabilization of the open complex might be explained by proposing that the K122R substitution primarily perturbs K122 possible interaction with G984, found only in the open complex, with minimal effects on the likely G985 interaction common to both conformations.

Interestingly, the Sui^–^ phenotype of K122R was found to be suppressed by the K91E substitution in the RRM, with the UUG:AUG ratio being returned to the WT level in the K122R K91E double mutant (Figure 5C, columns 2–3). K91 appears to interact with the rRNA residue U1011 in the open complex, and possibly with U1011, A1012 and G984 in the closed complex (Table 2). An intriguing possibility is that K91E alters the position of G984 in a manner that overcomes the perturbation of the adjacent rRNA residue G985 by the K122R substitution as the means of suppressing the Sui^–^ phenotype of the latter. The K91E substitution has no effect on cell growth alone or in combination with K122R (Supplementary Figure S12A, rows 3 and 5 versus 1), nor does it alter the UUG:AUG ratio in otherwise WT cells (Supplementary Figure S12E).

Finally, residue K346 in the eIF3b β-propeller approaches rRNA helix 44 (Supplementary Figure S11A), possibly contacting residue A1742 on the subunit interface only in the open conformation (Table 2). Consistent with selective destabilization of the open state and concomitant shift to the closed state, we found that K346E confers a weak Sui^–^ phenotype, increasing the UUG:AUG ratio by ≈2.2-fold (Figure 5C, columns 1 and 4).

(iii) Evidence that possible electrostatic repulsion of eIF3b β-propeller residues Asp397, Glu398, Asp297, and Glu308 with rRNA or eIF2γ on the 40S interface facilitate relocation of the eIF3b-3i-3g-3a-Cter module to the 40S solvent-exposed surface.

**Figure 6.**
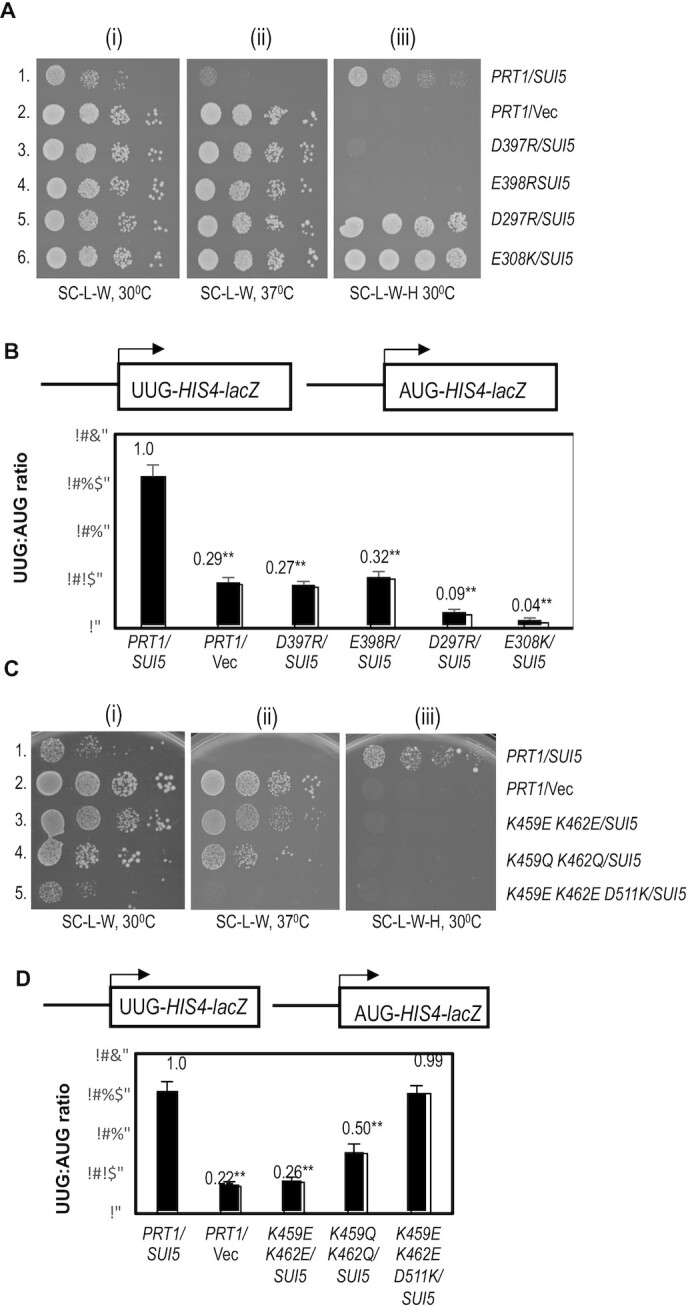
Effect of substitutions in the eIF3b β-propeller on the fidelity of start codon selection *in vivo* (A, B) Evidence that introducing excessively stable interactions of the eIF3b β-propeller with the 40S subunit or eIF2γ at the subunit interface increases discrimination against UUG start codons. (**A**) Serial dilutions of transformants of *P_GAL1_-PRT1 his4-301* strain HD3607 with the indicated *PRT1* alleles on low-copy (lc) *LEU2* plasmids and either single-copy (sc) *TRP1 SUI5* plasmid p4281 or empty vector YCplac22 (Vec) were spotted on synthetic complete medium lacking Leu and Trp (SC-L-W) or the same medium also lacking His (SC-L-W-H) and incubated at 30ºC or 37ºC for 3–5 days. (**B**) *HIS4-lacZ* UUG:AUG ratios were determined for strains in (A) exactly as in Figure 5B. (C, D) Evidence that weakening interactions of eIF3b β-propeller residues at the solvent-exposed surface of the 40S subunit increases discrimination against UUG start codons. (**C**) Serial dilutions of transformants of *P_GAL1_-PRT1 his4-301* strain HD3607 with the indicated *PRT1* alleles on low-copy (lc) *LEU2* plasmids and either single-copy (sc) *TRP1 SUI5* plasmid p4281 or empty vector YCplac22 (Vec) were spotted on synthetic complete medium lacking Leu and Trp (SC-L-W) or the same medium also lacking His (SC-L-W-H) and incubated at 30ºC or 37ºC for 3–5 days. (**D**) *HIS4-lacZ* UUG:AUG ratios were determined for strains in (C) exactly as in Figure 5B.

In addition to observing Ssu^–^ or Sui^–^ phenotypes for substitutions designed to weaken interactions of the eIF3b RRM or β-propeller on the subunit interface, we also examined substitutions that would be expected to stabilize interaction of the β-propeller at the interface, hypothesizing that such substitutions would impede relocation of the eIF3b/eIF3g/eIF3i module back to the solvent side of the 40S subunit. This would be expected to impair start codon recognition, particularly at near-cognate UUG codons, and confer an Ssu^–^ phenotype. Interestingly, the acidic eIF3b residues D397 and E398 are in proximity to helix 44 (Supplementary Figure S11) and may contact the phosphate backbone of rRNA residue G1743 exclusively in the closed complex (Table 2). Mutations that substitute both of these acidic residues with the basic residue Arg confer strong Ssu^–^ phenotypes, completely reversing the elevated UUG:AUG initiation ratio, Slg^–^ at 37°C, and His^+^ phenotypes conferred by *SUI5* (Figure 6A, rows 1 and 3–4; Figure 6B, columns 1 and 3–4). These findings are consistent with the idea that the adjacent acidic residues D397 and E398 exert electrostatic repulsion with rRNA residue G1743 in the closed complex that facilitates relocation of the WT eIF3b-3i-3g-3a-Cter module back to the 40S solvent side, and that introducing electrostatic attraction between the β-propeller and rRNA at this interface by the D397R and E398R substitution impedes this relocation and subsequent start codon selection.

Similar findings were made for basic substitutions of two acidic residues in the eIF3b β-propeller, D297 and E308, that are in proximity to acidic residues of eIF2γ in the closed complex, eIF2γ-D460 and eIF2γ-E506, respectively (Supplementary Figure S11 and Table 2). Replacing the eIF3b acidic residues with Arg (D297R) or Lys (E308K) each conferred Ssu^–^ phenotypes in *SUI5* cells, strongly suppressing the UUG:AUG initiation ratio (Figure 6B, columns rows 1, 5–6) and Slg^–^ at 37°C (Figure 6A, rows 1, 5–6) conferred by *SUI5*. Unexpectedly, neither mutation reverses the His^+^ phenotype of *SUI5* (Figure 5A, rows 1, 5–6), which might indicate a confounding effect of the mutations on histidine biosynthesis or utilization that we observed previously for certain other suppressors of *SUI5* (53). Notwithstanding this last complexity, the findings on D297R and E308K conform to the model that electrostatic repulsion between the eIF3b β-propeller and eIF2γ serves to facilitate relocation of the WT eIF3b-3i-3g-3a-Cter module to the solvent side of the 40S in a manner that can be impeded by replacing electrostatic repulsion with attraction at the β-propeller:eIF2γ interface. None of the basic substitutions in acidic residues D397, E398, D297, or E308 affected cell growth (Supplementary Figure S12A, rows 11–14 vs. 9) nor the UUG:AUG ratio in the absence of *SUI5* (Supplementary Figure S12D).

(iv) Evidence that possible interactions of eIF3b β-propeller residues Lys459 and Lys462 with rRNA on the solvent side of the 40S promote the relocation of the eIF3b-3i-3g-3a-Cter module.

The genetic results described above provide evidence that eIF3b contacts on the subunit interface are physiologically important for the fidelity of start codon selection. We hypothesized that eIF3b contacts on the solvent side of the 40S would also affect initiation by influencing whether the eIF3b-3i-3g-3a-Cter module can efficiently relocate back to this side of the 40S subunit to allow the completion of start codon selection. Examining the structure of yeast late initiation stage py48S-eIF5N PIC (PDB: 6FYX), in which the eIF3b-3i-3g-3a-Cter module is found on the solvent side of the 40S, revealed that eIF3b β-propeller residues K459 and K462 are in proximity to rRNA residues of h16 on the solvent side of the 40S, with K462 contacting the phosphate backbone of residue U498. In accordance with our hypothesis, the K459E/K462E double substitution in the β-propeller confers a strong Ssu^–^ phenotype in *SUI5* cells, suppressing the Slg^–^ at 37°C and His^+^ growth phenotypes and the elevated UUG:AUG ratio conferred by *SUI5* (Figure 6C, rows 1 and 3; Figure 6D, columns 1 and 3). K459E/K462E has no effect on the UUG:AUG initiation ratio in cells lacking *SUI5* (Supplementary Figure S12E); however, it does confer a moderate Slg^–^ phenotype at 30°C in otherwise WT cells (Supplementary Figure S12A, row 18), suggesting that this β-propeller:rRNA contact on the solvent side of the 40S might be important for the rate of initiation and not only its fidelity. Reasoning that the Glu substitutions of these eIF3b residues would not only eliminate electrostatic attraction but also introduce electrostatic repulsion with rRNA, we asked whether Gln rather than Glu substitutions have a less severe Ssu^–^ phenotype. Indeed, unlike K459E/K462E, the K459Q/K462Q substitution conferred no Slg^–^ in otherwise WT cells (Supplementary Figure S12A, row 20 versus 18 and 15) and suppression of the elevated UUG:AUG ratio conferred by *SUI5* was less complete for K459Q/K462Q compared to the K459E/K462E mutant (Figure 6D, rows 1 and 4 versus 3); although still strong enough to suppress the His^+^ and Slg^–^ phenotypes of *SUI5* (Figure 6C, rows 1 and 4). These findings support the idea that electrostatic attraction between eIF3b residues K459/K462 and rRNA residues on the solvent side of the 40S promote relocation of the eIF3b-3i-3g-3a-Cter module back to the solvent side to complete the process of start codon selection.

Interestingly, when K459E/K462E are combined with D511K, the ability of K459E/K462E to suppress the Slg^–^ at 37°C and elevated UUG:AUG initiation ratio conferred by *SUI5* is lost, as both the Slg^–^ at 37°C and elevated UUG initiation conferred by *SUI5* are reinstated in the triple mutant (Figure 6C, row 1 & 3 versus 5; Figure 6D, columns 1 and 3 versus 6). While it appears that the His^+^ phenotype of *SUI5* was not reinstated, the absence of a strong His^+^ phenotype in the *K459E/K462E/D511K* triple mutant in Figure 6C (row 5) can be explained by the strong Slg^–^ phenotypes conferred on + His medium by this triple mutation compared to the *K459E/K462E* double mutation (row 5 versus 3). While not in direct contact, D511 is in proximity to 18S rRNA residue C499 of h16 (PDB: 6FYX), such that introducing lysine at this position might strengthen the binding of the β-propeller to the solvent surface of the 40S and counteract the predicted effects of K459E/K462E in weakening this same interaction. If so, this would restore both efficient relocation of the eIF3b-3i-3g-3a-Cter module back to the solvent side of the 40S and efficient start codon selection, and thereby reverse the effects of K459E/K462E in reducing UUG initiation.

To assess the possible impact of the mutations on the abundance of the eIF3 complex, we conducted affinity purifications, directed against the His8 epitope attached to the eIF3b subunit, from whole cell extracts prepared from the WT or mutant strains described in Supplementary Figure S12. An isogenic strain expressing untagged WT eIF3b was examined in parallel as a control. We observed no effects of any of the substitutions on the steady-state abundance of the tagged eIF3b/Prt1 proteins or the amounts of co-purifying eIF3a/Tif32 or eIF3g/Tif35 proteins (Supplementary Figure S13). Hence, the phenotypes conferred by the eIF3b substitutions appear to arise from altered eIF3 function rather than reduced expression or integrity of eIF3. Taken together, the above genetic studies demonstrate that eIF3b interactions at the subunit interface of 40S modulate the fidelity of the start codon selection.

## CONCLUDING REMARKS

**Figure 7.**
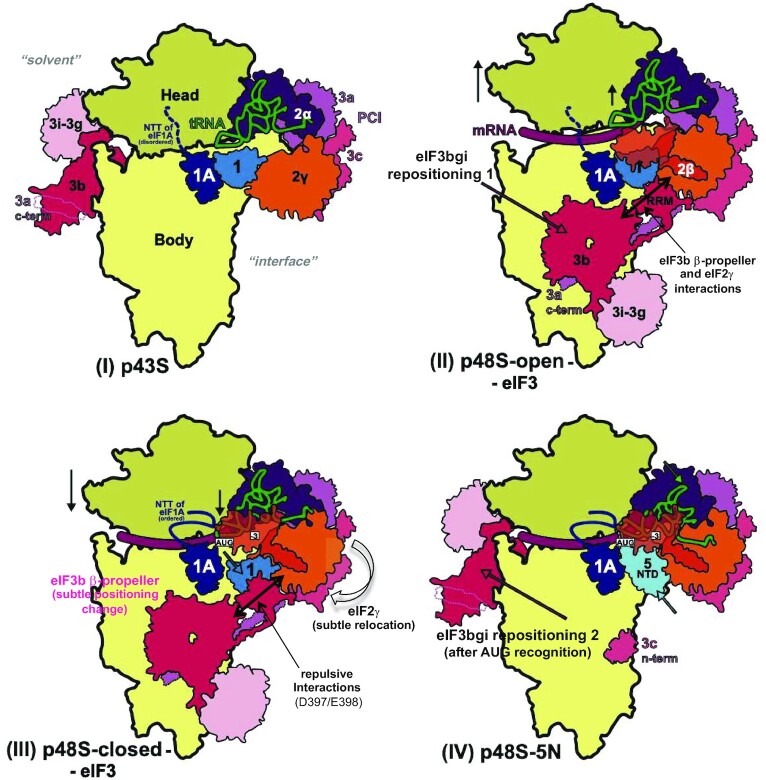
Model depicting reversible repositioning of the eIF3b/eIF3i/eIF3g/eIF3a-Cterm module between the solvent-exposed and subunit-interface surfaces of the 40S at the onset of scanning and following AUG recognition Schematics showing the two different locations observed for the eIF3b/eIF3i/eIF3g/eIF3a-Cterm module on the solvent or subunit surfaces of the 40S subunit in (I) p43S, (IV) py48S-eIF5N and (II/III) py48S-open-eIF3/py48S-closed-eIF3, respectively (modified from (9)). The red arrows in (II) and (IV) depict the direction of the movement of this eIF3 subcomplex from the solvent surface to the subunit interface upon 43S PIC attachment to mRNA and formation of the open, scanning conformation of the 48S PIC (Repositioning 1), and then back to the solvent surface after AUG recognition, complete accommodation of tRNA_i_ and eIF1 dissociation from the 48S complex (Repositioning 2). Also indicated are upward or downward movements of the 40S head relative to the body (single-headed black arrows), additional interactions (two-headed black arrows) and subtle conformational changes (grey arrows or red type) of different eIF3 elements, tRNA, the 40S head and eIF2γ at the subunit interface accompanying transition from the open to closed states of the 48S complex on AUG recognition (II)-III); followed by repositioning of the eIF3b/eIF3i/eIF3g/eIF3a-Cterm module (eIF3bgi) to the solvent side of the 40S following AUG recognition, eIF1 dissociation and replacement of eIF1 with the eIF5 NTD near the P-site (IV).

The structures of py48S-open-eIF3 PIC and py48S-closed-eIF3 presented here show that the β-propeller and RRM of eIF3b relocate at the 40S subunit interface. eIF3b adopts a similar conformation in both complexes with subtle differences in its interactions with 48S PIC components at the subunit interface in the open versus closed states (Figure 4E, Movie 1). eIF3i is also observed in the new structures; however, its β-propeller is weakly attached to eIF3b and does not make any direct contacts with the 40S subunit. This stands in contrast to a model proposed recently for the rearrangement of eIF3 during translation initiation wherein the β-propellers of eIF3b and eIF3i relocate independently to the subunit interface at different stages of the process (13). This model was based on a model of a mammalian 48S late-stage IC in which the β-propeller of eIF3i was positioned at the GTPase-binding site on the subunit interface (13). However, as this density was later re-interpreted as the ABCE1 protein (15), the model proposed by Simonetti *et al.* (13) now seems unlikely. Instead, we propose an alternative model in which the β-propellers of eIF3b and eIF3i relocate together from the solvent interface of the 43S PIC to the subunit interface after mRNA binding or in early stages of codon:anticodon recognition, with only the eIF3b propeller contacting the 40S subunit in both states, and thereafter these domains relocate together back to their original positions on the solvent interface after the release of eIF1 and its replacement by the eIF5 N-terminal domain at the subunit interface (9) (Figure 7 and Movie 3). Recently, in a human 48S scanning-competent PIC, the eIF3b-3i-3g-3a-Cter was observed in its canonical position at the solvent surface (43), as we observed previously in the yeast late-stage py48S-eIF5N complex (9). We reason that the difference in eIF3b-3i-3g-3a-Cter in the human PIC and our work could represent PICs at different stages of the initiation pathway; or could reflect relatively greater stability of the eIF3b-3i-3g-3a-Cter module on the subunit interface in yeast.

A key consequence of the relocation of eIF3b from the solvent interface in the 43S PIC to the subunit interface in our py48S-open-eIF3 and py48S-closed-eIF3 structures is to bring the eIF3b β-propeller in contact with eIF2γ, and the eIF3b RRM in contact with eIF1 and the eIF3c-NTD. We provided independent evidence supporting this relocation by demonstrating that the eIF3c-NTD can interact directly with the eIF3b RRM in addition to its known interaction with eIF1, and further showed that the eIF3c-NTD/eIF3b RRM interaction in solution was weakened by substituting RRM residues predicted to contact eIF3c-NTD in our structures. Secondly, we demonstrated that genetic perturbation of predicted interactions of eIF3b with the 40S or other eIFs at the subunit interface, including a substitution found to weaken eIF3c-NTD/eIF3b RRM association in solution, alter the frequency of initiation at UUG codons *in vivo*, indicating that eIF3b contacts on the subunit interface are physiologically important for the fidelity of start codon selection.

Based on previous studies (1), the modest Sui^–^ phenotypes conferred by substitutions in the RRM (K122R) or β-propeller (K346E) suggest that predicted interactions of these residues with rRNA at the subunit interface preferentially stabilize the open conformation, such that weakening them allows inappropriate rearrangement to the closed state at UUG codons. The much stronger Ssu^–^ phenotypes conferred by the β-propeller substitutions K147A and K152E identifies a more critical role for these eIF3b contacts with the eIF3c (K147A) or rRNA (K152E) in achieving or stabilizing the closed conformation of the PIC, as disrupting them shifts the system to the open conformation to impair UUG selection (Supplementary Figure S11). The fact that perturbing different eIF3b contacts at the subunit interface can have opposing effects on fidelity could be explained if the affected interactions are restricted to one conformation, owing to the subtle changes in the position of the eIF3b-3i-3g-3a-Cter module between the open and closed conformations of the scanning PIC (Movies 1 and 3 and Figure 7(II)-(III)), or if they are relatively more important in stabilizing one of the two conformations.

Strong Ssu^–^ phenotypes were also observed for eIF3b β-propeller substitutions expected to impede relocation of the eIF3b-3i-3g-3a-Cter module from the interface back to the solvent side of the 40S subunit, by either of two mechanisms. The first involves β-propeller substitutions that diminish electrostatic repulsion between eIF3b and rRNA (D397R and E398R) or eIF3b and eIF2γ (D297R and E308K) at the subunit interface, which should confer an abnormally stable interaction of the eIF3b-3i-3g-3a-Cter module at the subunit interface (Supplementary Figure S11). The second mechanism involves a double substitution predicted to disrupt eIF3b interaction with rRNA specifically on the solvent side of the 40S, K459E/K462E, which should selectively weaken association of the eIF3b-3i-3g-3a-Cter module on the solvent surface (Supplementary Figure S11). The repulsive interactions between eIF3b residues D397/E398 and rRNA at the subunit interface of wild-type PICs, which should favour release of the eIF3b-3i-3g-3a-Cter module for its relocation back to the solvent surface on AUG recognition (perturbed by the first mechanism), appear to exist only in the closed conformation of the PIC, owing to downward movement of the 40S head in the transition from open to closed states (Figure 7 (III) versus (II)).

Together, our genetic findings suggest that efficient start codon recognition, and utilization of near-cognate UUG codons in particular, depends not only on interactions of eIF3b at the subunit interface that stabilize the closed conformation of the PIC, but also on the timely dissolution of these interactions to permit relocation of the eIF3b-3i-3g-3a-Cter module back to the solvent side of the 40S subunit following AUG recognition. As such, different eIF3b substitutions predicted to either selectively weaken its interactions on the subunit interface, to render these interactions excessively stable, or to selectively diminish its interactions on the solvent side of the 40S subunit, all have the same consequence of disfavouring initiation at near-cognate UUG codons.

The relocation of the eIF3b-3i-3g-3a-Cter module from the 40S subunit interface back to the solvent side of the 40S (Repositioning 2 (IV), Figure 7) may facilitate dissociation of eIF1 from the 40S interface owing to loss of eIF1 interactions with the eIF3b RRM, or promote subsequent dissociation of eIF2 from Met-tRNA_i_ owing to loss of eIF2γ contacts with the eIF3b β-propeller occurring at the subunit interface. This relocation should also be required for joining of the 60S subunit, which requires extensive interactions with the 40S interface, some of which would be sterically hindered by the presence of the eIF3b-3i-3g-3a-Cter module at this position.

eIF3 is involved in nearly all steps of initiation, including stimulating the binding of TC and other eIFs to the 40S ribosomal subunit; the attachment of the 43S complex to mRNA; the subsequent scanning along mRNA to reach the start AUG codon; and finally, prevention of the joining of small and large ribosomal subunits prior to start codon recognition (1,4,6). However, little is known about the molecular basis of these various functions of eIF3 and the role of each subunit in executing them. In particular, there was no understanding of the functional significance of the conformation of eIF3 in the PIC with its peripheral eIF3b-3i-3g-3a-Cter module positioned on the subunit interface, as described in detail here. Overall, the structural models of eIF3 and genetic analysis presented in this study suggest that distinct eIF3b interactions on the subunit interface play crucial roles in stabilizing one or more features of the closed conformation, or in modulating relocation of the eIF3b-3g-3i module back to the solvent side of the 40S to license one or more late steps of the initiation pathway following recognition of the start codon.

## DATA AVAILABILITY

Two maps have been deposited in the EMDB with accession codes EMDB: 0057 and EMDB: 0058, for the py48S-open-eIF3 and py48S-closed-eIF3 maps, respectively. Two atomic coordinate models have been deposited in the PDB with accession codes PDB: 6GSM and PDB: 6GSN, for the py48S-open-eIF3 and py48S-closed-eIF3 models, respectively. These models replace previous PDB: 3JAQ and PDB: 3JAP models (12) and are also linked to previous published maps EMDB: 3050 and EMDB: 3049, respectively (12).

## Supplementary Material

gkab908_Supplemental_Files

## References

[1] Hinnebusch A.G. Structural Insights into the mechanism of scanning and start codon recognition in eukaryotic translation initiation. Trends Biochem. Sci. 2017; 42:589–611. 28442192 10.1016/j.tibs.2017.03.004

[2] Pelletier J. , Sonenberg N. The organizing principles of eukaryotic ribosome recruitment. Annu. Rev. Biochem. 2019; 88:307–335. 31220979 10.1146/annurev-biochem-013118-111042

[3] Mishra R.K. , Datey A., Hussain T. mRNA recruiting eIF4 factors involved in protein synthesis and its regulation. Biochemistry. 2019; 59:34–46. 31765127 10.1021/acs.biochem.9b00788

[4] Hinnebusch A.G. The scanning mechanism of eukaryotic translation initiation. Annu. Rev. Biochem. 2014; 83:779–812. 24499181 10.1146/annurev-biochem-060713-035802

[5] Aylett C.H. , Ban N. Eukaryotic aspects of translation initiation brought into focus. Philos. Trans. R. Soc. Lond. B Biol. Sci. 2017; 372:20160186. 28138072 10.1098/rstb.2016.0186 PMC5311930

[6] Valášek L.S. , Zeman J., Wagner S., Beznosková P., Pavlíková Z., Mohammad M.P., Hronová V., Herrmannová A., Hashem Y., Gunišová S. Embraced by eIF3: structural and functional insights into the roles of eIF3 across the translation cycle. Nucleic Acids Res. 2017; 45:10948–10968. 28981723 10.1093/nar/gkx805 PMC5737393

[7] Hashem Y. , des Georges A., Dhote V., Langlois R., Liao H.Y., Grassucci R.A., Hellen C.U., Pestova T.V., Frank J. Structure of the mammalian ribosomal 43S preinitiation complex bound to the scanning factor DHX29. Cell. 2013; 153:1108–1119. 23706745 10.1016/j.cell.2013.04.036 PMC3730827

[8] Aylett C.H. , Boehringer D., Erzberger J.P., Schaefer T., Ban N. Structure of a yeast 40S-eIF1-eIF1A-eIF3-eIF3j initiation complex. Nat. Struct. Mol. Biol. 2015; 22:269–271. 25664723 10.1038/nsmb.2963

[9] Llácer J.L. , Hussain T., Saini A.K., Nanda J.S., Kaur S., Gordiyenko Y., Kumar R., Hinnebusch A.G., Lorsch J.R., Ramakrishnan V. Translational initiation factor eIF5 replaces eIF1 on the 40S ribosomal subunit to promote start-codon recognition. Elife. 2018; 7:e39273. 30475211 10.7554/eLife.39273 PMC6298780

[10] des Georges A. , Dhote V., Kuhn L., Hellen C.U., Pestova T.V., Frank J., Hashem Y. Structure of mammalian eIF3 in the context of the 43S preinitiation complex. Nature. 2015; 525:491–495. 26344199 10.1038/nature14891 PMC4719162

[11] Eliseev B. , Yeramala L., Leitner A., Karuppasamy M., Raimondeau E., Huard K., Alkalaeva E., Aebersold R., Schaffitzel C. Structure of a human cap-dependent 48S translation pre-initiation complex. Nucleic Acids Res. 2018; 46:2678–2689. 29401259 10.1093/nar/gky054 PMC5861459

[12] Llácer J.L. , Hussain T., Marler L., Aitken C.E., Thakur A., Lorsch J.R., Hinnebusch A.G., Ramakrishnan V. Conformational differences between open and closed states of the eukaryotic translation initiation complex. Mol. Cell. 2015; 59:399–412. 26212456 10.1016/j.molcel.2015.06.033 PMC4534855

[13] Simonetti A. , Brito Querido J., Myasnikov A.G., Mancera-Martinez E., Renaud A., Kuhn L., Hashem Y. eIF3 peripheral subunits rearrangement after mRNA binding and start-codon recognition. Mol. Cell. 2016; 63:206–217. 27373335 10.1016/j.molcel.2016.05.033

[14] Heuer A. , Gerovac M., Schmidt C., Trowitzsch S., Preis A., Kötter P., Berninghausen O., Becker T., Beckmann R., Tampé R. Structure of the 40S-ABCE1 post-splitting complex in ribosome recycling and translation initiation. Nat. Struct. Mol. Biol. 2017; 24:453–460. 28368393 10.1038/nsmb.3396

[15] Mancera-Martínez E. , Brito Querido J., Valasek L.S., Simonetti A., Hashem Y. ABCE1: a special factor that orchestrates translation at the crossroad between recycling and initiation. RNA Biol. 2017; 14:1279–1285. 28498001 10.1080/15476286.2016.1269993 PMC5711452

[16] Fernández I.S. , Bai X.C., Murshudov G., Scheres S.H., Ramakrishnan V. Initiation of translation by cricket paralysis virus IRES requires its translocation in the ribosome. Cell. 2014; 157:823–831. 24792965 10.1016/j.cell.2014.04.015 PMC4017093

[17] Acker M.G. , Kolitz S.E., Mitchell S.F., Nanda J.S., Lorsch J.R. Reconstitution of yeast translation initiation. Methods Enzymol. 2007; 430:111–145. 17913637 10.1016/S0076-6879(07)30006-2

[18] Mitchell S.F. , Walker S.E., Algire M.A., Park E.H., Hinnebusch A.G., Lorsch J.R. The 5′-7-methylguanosine cap on eukaryotic mRNAs serves both to stimulate canonical translation initiation and to block an alternative pathway. Mol. Cell. 2010; 39:950–962. 20864040 10.1016/j.molcel.2010.08.021 PMC2945613

[19] Li X. , Mooney P., Zheng S., Booth C.R., Braunfeld M.B., Gubbens S., Agard D.A., Cheng Y. Electron counting and beam-induced motion correction enable near-atomic-resolution single-particle cryo-EM. Nat. Methods. 2013; 10:584–590. 23644547 10.1038/nmeth.2472 PMC3684049

[20] Zhang K. Gctf: Real-time CTF determination and correction. J. Struct. Biol. 2016; 193:1–12. 26592709 10.1016/j.jsb.2015.11.003 PMC4711343

[21] Scheres S.H. RELION: implementation of a Bayesian approach to cryo-EM structure determination. J. Struct. Biol. 2012; 180:519–530. 23000701 10.1016/j.jsb.2012.09.006 PMC3690530

[22] Scheres S.H. Semi-automated selection of cryo-EM particles in RELION-1.3. J. Struct. Biol. 2015; 189:114–122. 25486611 10.1016/j.jsb.2014.11.010 PMC4318617

[23] Tang G. , Peng L., Baldwin P.R., Mann D.S., Jiang W., Rees I., Ludtke S.J. EMAN2: an extensible image processing suite for electron microscopy. J. Struct. Biol. 2007; 157:38–46. 16859925 10.1016/j.jsb.2006.05.009

[24] Bai X.C. , Fernandez I.S., McMullan G., Scheres S.H. Ribosome structures to near-atomic resolution from thirty thousand cryo-EM particles. Elife. 2013; 2:e00461. 23427024 10.7554/eLife.00461 PMC3576727

[25] Scheres S.H. , Chen S. Prevention of overfitting in cryo-EM structure determination. Nat. Methods. 2012; 9:853–854. 22842542 10.1038/nmeth.2115 PMC4912033

[26] Rosenthal P.B. , Henderson R. Optimal determination of particle orientation, absolute hand, and contrast loss in single-particle electron cryomicroscopy. J. Mol. Biol. 2003; 333:721–745. 14568533 10.1016/j.jmb.2003.07.013

[27] Kucukelbir A. , Sigworth F.J., Tagare H.D. Quantifying the local resolution of cryo-EM density maps. Nat. Methods. 2014; 11:63–65. 24213166 10.1038/nmeth.2727 PMC3903095

[28] Pettersen E.F. , Goddard T.D., Huang C.C., Couch G.S., Greenblatt D.M., Meng E.C., Ferrin T.E. UCSF Chimera – a visualization system for exploratory research and analysis. J. Comput. Chem. 2004; 25:1605–1612. 15264254 10.1002/jcc.20084

[29] Emsley P. , Lohkamp B., Scott W.G., Cowtan K. Features and development of Coot. Acta Crystallogr. D. Biol. Crystallogr. 2010; 66:486–501. 20383002 10.1107/S0907444910007493 PMC2852313

[30] Ramlaul K. , Palmer C.M., Aylett C.H.S. A local agreement filtering algorithm for transmission EM reconstructions. J. Struct. Biol. 2019; 205:30–40. 30502495 10.1016/j.jsb.2018.11.011 PMC6351148

[31] Reibarkh M. , Yamamoto Y., Singh C.R., del Rio F., Fahmy A., Lee B., Luna R.E., Ii M., Wagner G., Asano K. et al. Eukaryotic initiation factor (eIF) 1 carries two distinct eIF5-binding faces important for multifactor assembly and AUG selection. J. Biol. Chem. 2008; 38:1094–1103. 10.1074/jbc.M708155200 17974565

[32] Chiu W.L. , Wagner S., Herrmannová A., Burela L., Zhang F., Saini A.K., Valásek L., Hinnebusch A.G. The C-terminal region of eukaryotic translation initiation factor 3a (eIF3a) promotes mRNA recruitment, scanning, and, together with eIF3j and the eIF3b RNA recognition motif, selection of AUG start codons. Mol. Cell. Biol. 2010; 30:4415–4434. 20584985 10.1128/MCB.00280-10 PMC2937525

[33] Khoshnevis S. , Gunišová S., Vlčková V., Kouba T., Neumann P., Beznosková P., Ficner R., Valášek L.S. Structural integrity of the PCI domain of eIF3a/TIF32 is required for mRNA recruitment to the 43S pre-initiation complexes. Nucleic Acids Res. 2014; 42:4123–4139. 24423867 10.1093/nar/gkt1369 PMC3973348

[34] Szamecz B. , Rutkai E., Cuchalová L., Munzarová V., Herrmannová A., Nielsen K.H., Burela L., Hinnebusch A.G., Valásek L. eIF3a cooperates with sequences 5′ of uORF1 to promote resumption of scanning by post-termination ribosomes for reinitiation on GCN4 mRNA. Genes Dev. 2008; 22:2414–2425. 18765792 10.1101/gad.480508 PMC2532924

[35] Brown A. , Long F., Nicholls R.A., Toots J., Emsley P., Murshudov G. Tools for macromolecular model building and refinement into electron cryo-microscopy reconstructions. Acta Crystallogr. D. Biol. Crystallogr. 2015; 71:136–153. 25615868 10.1107/S1399004714021683 PMC4304694

[36] Chen V.B. , Arendall W.B., Headd J.J., Keedy D.A., Immormino R.M., Kapral G.J., Murray L.W., Richardson J.S., Richardson D.C. MolProbity: all-atom structure validation for macromolecular crystallography. Acta Crystallogr. D. Biol. Crystallogr. 2010; 66:12–21. 20057044 10.1107/S0907444909042073 PMC2803126

[37] Amunts A. , Brown A., Bai X.C., Llácer J.L., Hussain T., Emsley P., Long F., Murshudov G., Scheres S.H.W., Ramakrishnan V. Structure of the yeast mitochondrial large ribosomal subunit. Science. 2014; 343:1485–1489. 24675956 10.1126/science.1249410 PMC4046073

[38] Krissinel E. , Henrick K. Inference of macromolecular assemblies from crystalline state. J. Mol. Biol. 2007; 372:774–797. 17681537 10.1016/j.jmb.2007.05.022

[39] DeLano W.L. 2006; The PyMOL Molecular Graphics System, Version 2.0 Schrödinger, LLC.

[40] Cross F.R. Marker swap’ plasmids: convenient tools for budding yeast molecular genetics. Yeast. 1997; 13:647–653. 9200814 10.1002/(SICI)1097-0061(19970615)13:7<647::AID-YEA115>3.0.CO;2-#

[41] Moehle C.M. , Hinnebusch A.G. Association of RAP1 binding sites with stringent control of ribosomal protein gene transcription in Saccharomyces cerevisiae. Mol. Cell. Biol. 1991; 11:2723–2735. 2017175 10.1128/mcb.11.5.2723-2735.1991 PMC360042

[42] Nielsen K.H. , Valásek L. In vivo deletion analysis of the architecture of a multiprotein complex of translation initiation factors. Methods Enzymol. 2007; 431:15–32. 17923228 10.1016/S0076-6879(07)31002-1

[43] Brito Querido J. , Sokabe M., Kraatz S., Gordiyenko Y., Skehel J.M., Fraser C.S., Ramakrishnan V. Structure of a human 48. Science. 2020; 369:1220–1227. 32883864 10.1126/science.aba4904 PMC7116333

[44] Hussain T. , Llácer J.L., Fernández I.S., Munoz A., Martin-Marcos P., Savva C.G., Lorsch J.R., Hinnebusch A.G., Ramakrishnan V. Structural changes enable start codon recognition by the eukaryotic translation initiation complex. Cell. 2014; 159:597–607. 25417110 10.1016/j.cell.2014.10.001 PMC4217140

[45] Thakur A. , Hinnebusch A.G. eIF1 Loop 2 interactions with Met-tRNA. Proc. Natl. Acad. Sci. U.S.A. 2018; 115:E4159–E4168. 29666249 10.1073/pnas.1800938115 PMC5939108

[46] Hussain T. , Llácer J.L., Wimberly B.T., Kieft J.S., Ramakrishnan V. Large-scale movements of IF3 and tRNA during Bacterial translation initiation. Cell. 2016; 167:133–144. 27662086 10.1016/j.cell.2016.08.074 PMC5037330

[47] Herrmannová A. , Daujotyte D., Yang J.C., Cuchalová L., Gorrec F., Wagner S., Dányi I., Lukavsky P.J., Valásek L.S. Structural analysis of an eIF3 subcomplex reveals conserved interactions required for a stable and proper translation pre-initiation complex assembly. Nucleic Acids Res. 2012; 40:2294–2311. 22090426 10.1093/nar/gkr765 PMC3300007

[48] Kashiwagi K. , Yokoyama T., Nishimoto M., Takahashi M., Sakamoto A., Yonemochi M., Shirouzu M., Ito T. Structural basis for eIF2B inhibition in integrated stress response. Science. 2019; 364:495–499. 31048492 10.1126/science.aaw4104

[49] Kenner L.R. , Anand A.A., Nguyen H.C., Myasnikov A.G., Klose C.J., McGeever L.A., Tsai J.C., Miller-Vedam L.E., Walter P., Frost A. eIF2B-catalyzed nucleotide exchange and phosphoregulation by the integrated stress response. Science. 2019; 364:491–495. 31048491 10.1126/science.aaw2922 PMC6601628

[50] Huang H.K. , Yoon H., Hannig E.M., Donahue T.F. GTP hydrolysis controls stringent selection of the AUG start codon during translation initiation in Saccharomyces cerevisiae. Genes Dev. 1997; 11:2396–2413. 9308967 10.1101/gad.11.18.2396 PMC316512

[51] Martin-Marcos P. , Nanda J.S., Luna R.E., Zhang F., Saini A.K., Cherkasova V.A., Wagner G., Lorsch J.R., Hinnebusch A.G. Enhanced eIF1 binding to the 40S ribosome impedes conformational rearrangements of the preinitiation complex and elevates initiation accuracy. RNA. 2014; 20:150–167. 24335188 10.1261/rna.042069.113 PMC3895268

[52] Martin-Marcos P. , Cheung Y.N., Hinnebusch A.G. Functional elements in initiation factors 1, 1A, and 2β discriminate against poor AUG context and non-AUG start codons. Mol. Cell. Biol. 2011; 31:4814–4831. 21930786 10.1128/MCB.05819-11 PMC3232919

[53] Saini A.K. , Nanda J.S., Martin-Marcos P., Dong J., Zhang F., Bhardwaj M., Lorsch J.R., Hinnebusch A.G. Eukaryotic translation initiation factor eIF5 promotes the accuracy of start codon recognition by regulating Pi release and conformational transitions of the preinitiation complex. Nucleic Acids Res. 2014; 42:9623–9640. 25114053 10.1093/nar/gku653 PMC4150770

[54] Martin-Marcos P. , Nanda J., Luna R.E., Wagner G., Lorsch J.R., Hinnebusch A.G. β-Hairpin loop of eukaryotic initiation factor 1 (eIF1) mediates 40 S ribosome binding to regulate initiator tRNA(Met) recruitment and accuracy of AUG selection in vivo. J. Biol. Chem. 2013; 288:27546–27562. 23893413 10.1074/jbc.M113.498642 PMC3779751

