# Supplementary material for: Large-scale movement of eIF3 domains during translation initiation modulate start codon selection
Source: Nucleic Acids Res. 2021 Oct 14;49(20):11491–511. doi: 10.1093/nar/gkab908 (PMC8599844; doi:10.1093/nar/gkab908)
Supplement: gkab908_Supplemental_Files [file gkab908_supplemental_files.zip › Supplementary_all.pdf]

Figure S1

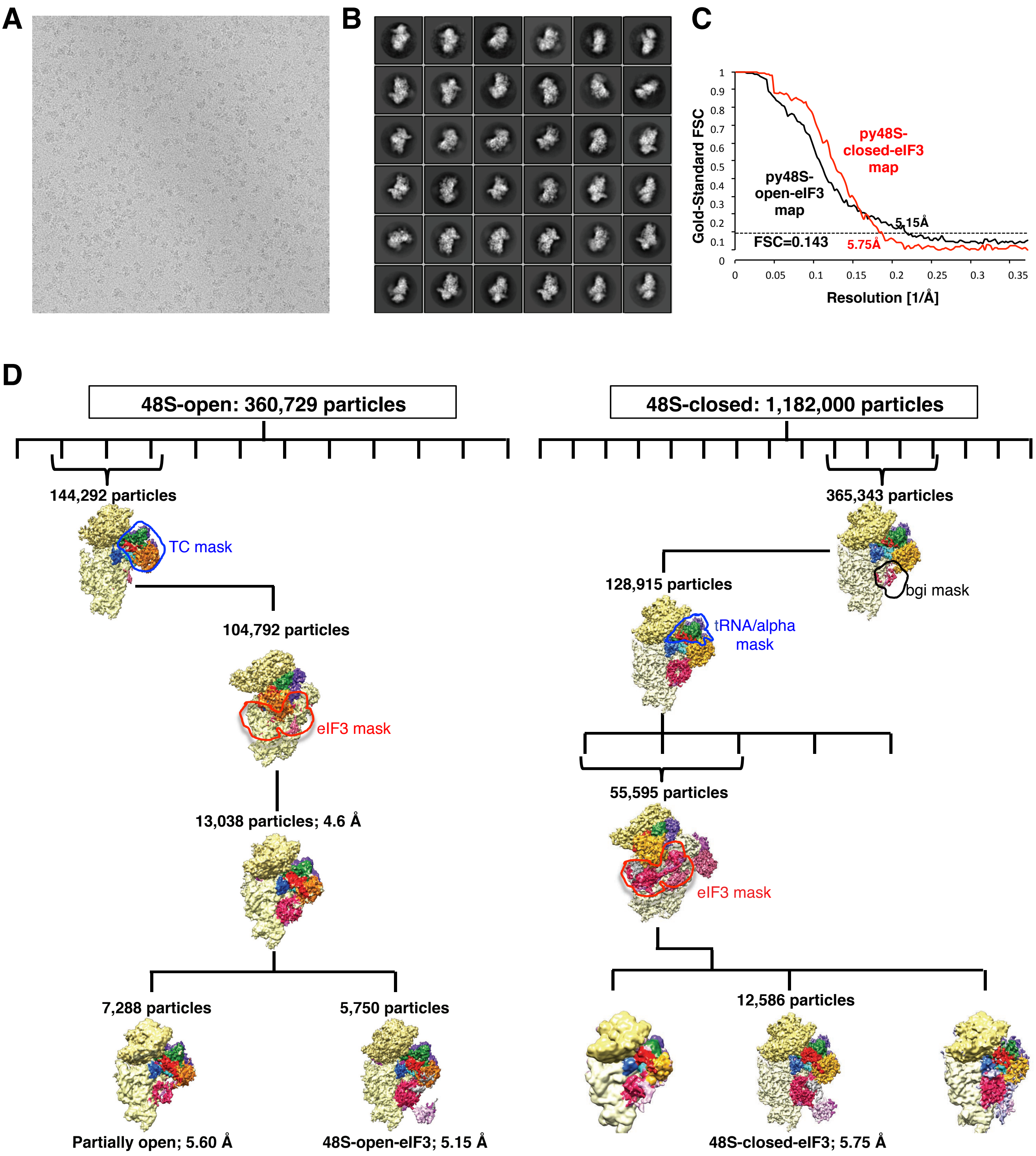
A
B
C
Gold-Standard FSC
Resolution [1/Å]
py48S-closed-eIF3 map
py48S-open-eIF3 map
5.15Å
5.75Å
FSC=0.143
D
48S-open: 360,729 particles
48S-closed: 1,182,000 particles
144,292 particles
TC mask
104,792 particles
eIF3 mask
13,038 particles; 4.6 Å
7,288 particles
5,750 particles
Partially open; 5.60 Å
48S-open-eIF3; 5.15 Å
365,343 particles
bgi mask
128,915 particles
tRNA/alpha mask
55,595 particles
eIF3 mask
12,586 particles
48S-closed-eIF3; 5.75 Å

Figure S2

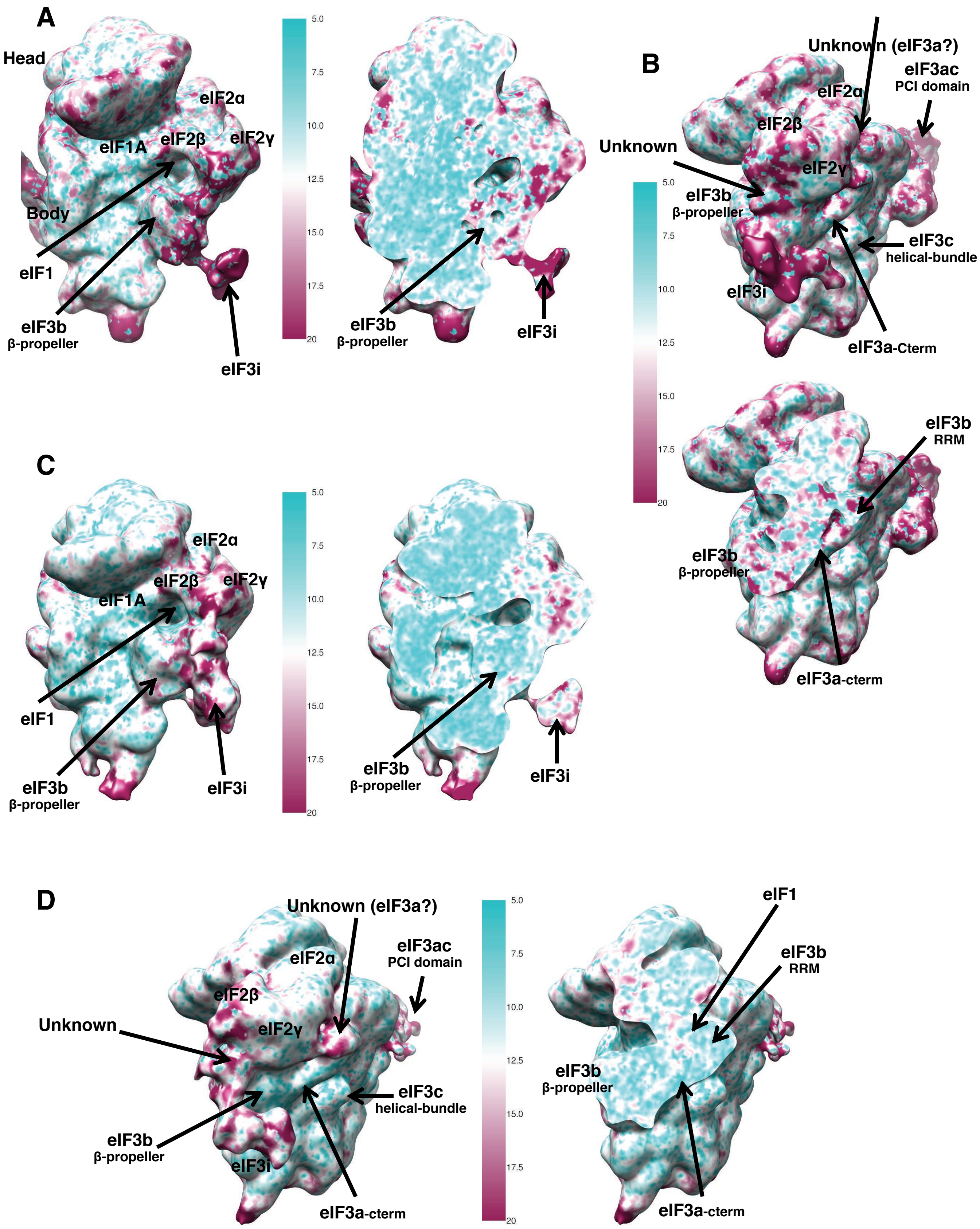
A
Head
eIF2α
eIF1A
eIF2β
eIF2γ
Body
eIF1
eIF3b
β-propeller
eIF3i
5.0
7.5
10.0
12.5
15.0
17.5
20
B
Unknown (eIF3a?)
eIF3ac
PCI domain
eIF2α
eIF2β
Unknown
eIF2γ
eIF3b
β-propeller
eIF3c
helical-bundle
eIF3i
eIF3a-Cterm
eIF3b
RRM
eIF3b
β-propeller
eIF3a-cterm
C
eIF2α
eIF2β
eIF2γ
eIF1A
eIF1
eIF3b
β-propeller
eIF3i
D
Unknown (eIF3a?)
eIF3ac
PCI domain
eIF2α
eIF2β
Unknown
eIF2γ
eIF3c
helical-bundle
eIF3b
β-propeller
eIF3i
eIF3a-cterm
eIF1
eIF3b
RRM
eIF3b
β-propeller
eIF3a-cterm

Figure S3

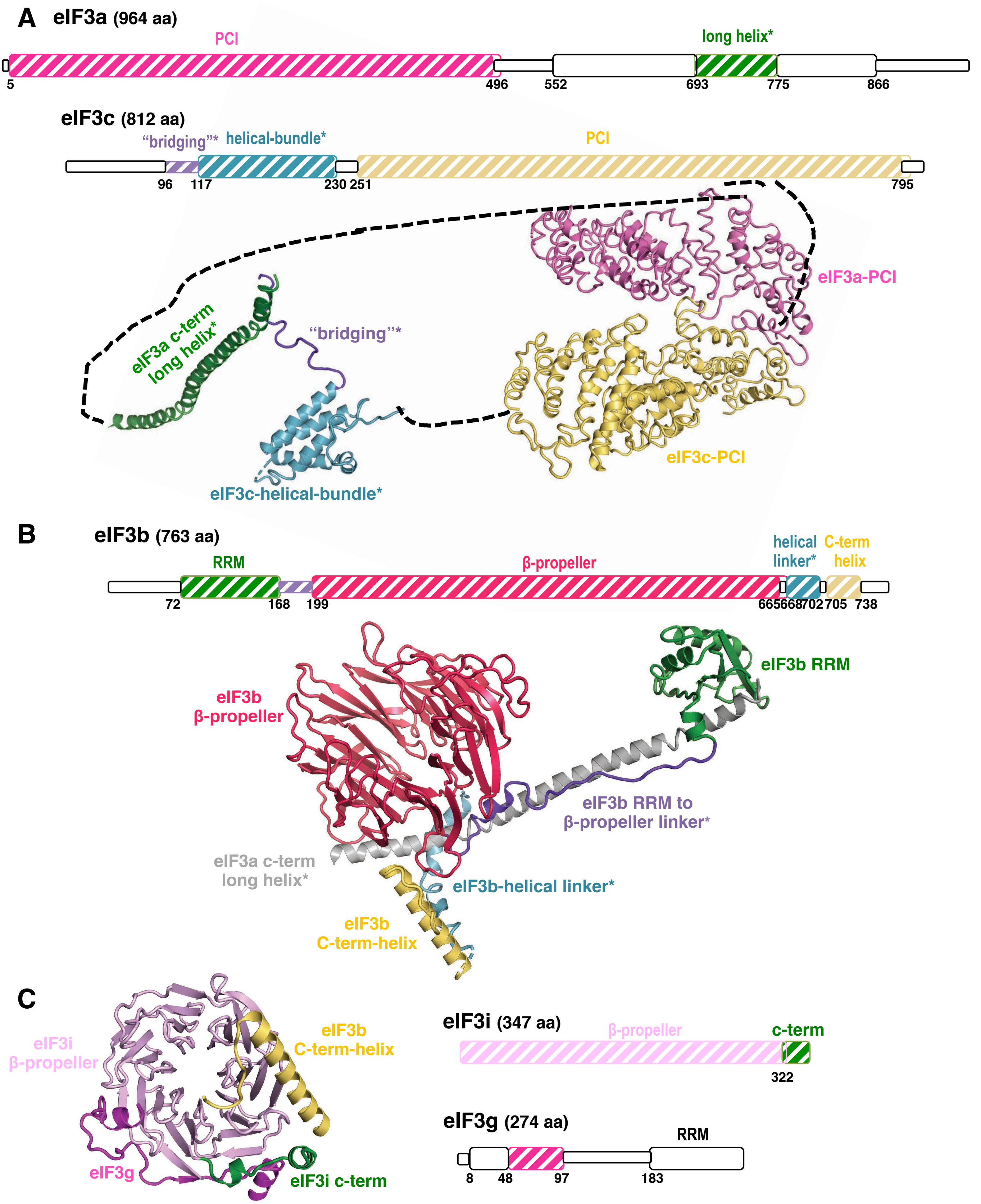

* Built as polyalanines

Figure S4

A eIF3c NTD (1-250)

```
MSRFFSSNYEYDVASSSSEEDLLSSSEEDLLSSSSSESELDQESDDSFFNESESEADVDSDDSDAKPYGPDWFKKSEFRKQSE 83

                  98          109     117                 133      143                 156
GGGSNKFLKSSNYDSSDEESDEEDGKKVVKSAKEKLLDEMQDVYNKISQAENSDDWLTISNEFDLISRLLVRAQQQNWGTPN 165

166                                185 189                        204207           216
IFIKVVAQVEDAVNNTQQADLKNKAVARAYNTTKQRVKKVSRENEDSMAKFRNDPESFDKEPTADLDISANGFTISSSQGNDQAV 250
```

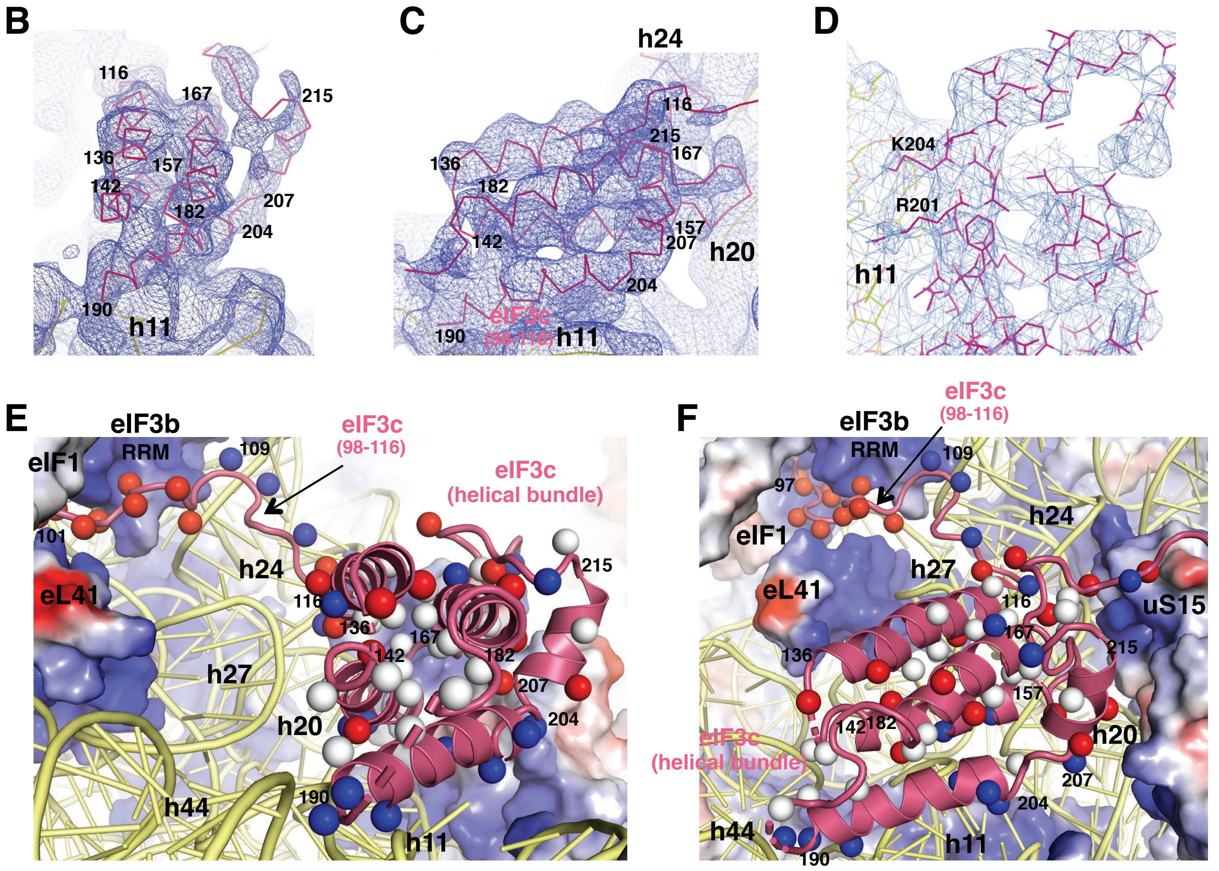

G eIF3a CTD (496-964)

```
FDIFASTASKEVSEEENTEPEVQEEKEETDEALGPQETEDGEEKEEESDPVIIRNSYIHNKLLELSNVLHDVDSFNNASY 575

MEKVRIARETLIKKNKDDLEKISKIVDERVKRSQEQKQKHMEHAALHAEQDAEVRQQRILEEKAAIEAKLEEEAHRRLIEKK 657

KREFEAIKEREITKMITEVNAKGHVYIDPNEAKSLDLDTIKQVIIAEVSKNKSELESRMEYAMKKLDHTERALRKVELPLLQK 740

EVDKLQETDTANYEAMKKKIVDAAKAEYEARMADRKNLVMVYDDYLKFKEHVSGTKESELAAIRNQKKAELEAAKKARIEEV 822

RKRRYEEAIARRKEEIANAERQKRAQELAEATRKQREIEEAAAKKSTPYSFRAGNREPPSTPSTLPKATVSPDKAKLDMIAQK 905

QREMEEAIEQRLAGRTAGGSSPATPATPATPATPTPSSGPKKMTMAEKLRAKRLAKGGR 964
```

Figure S5

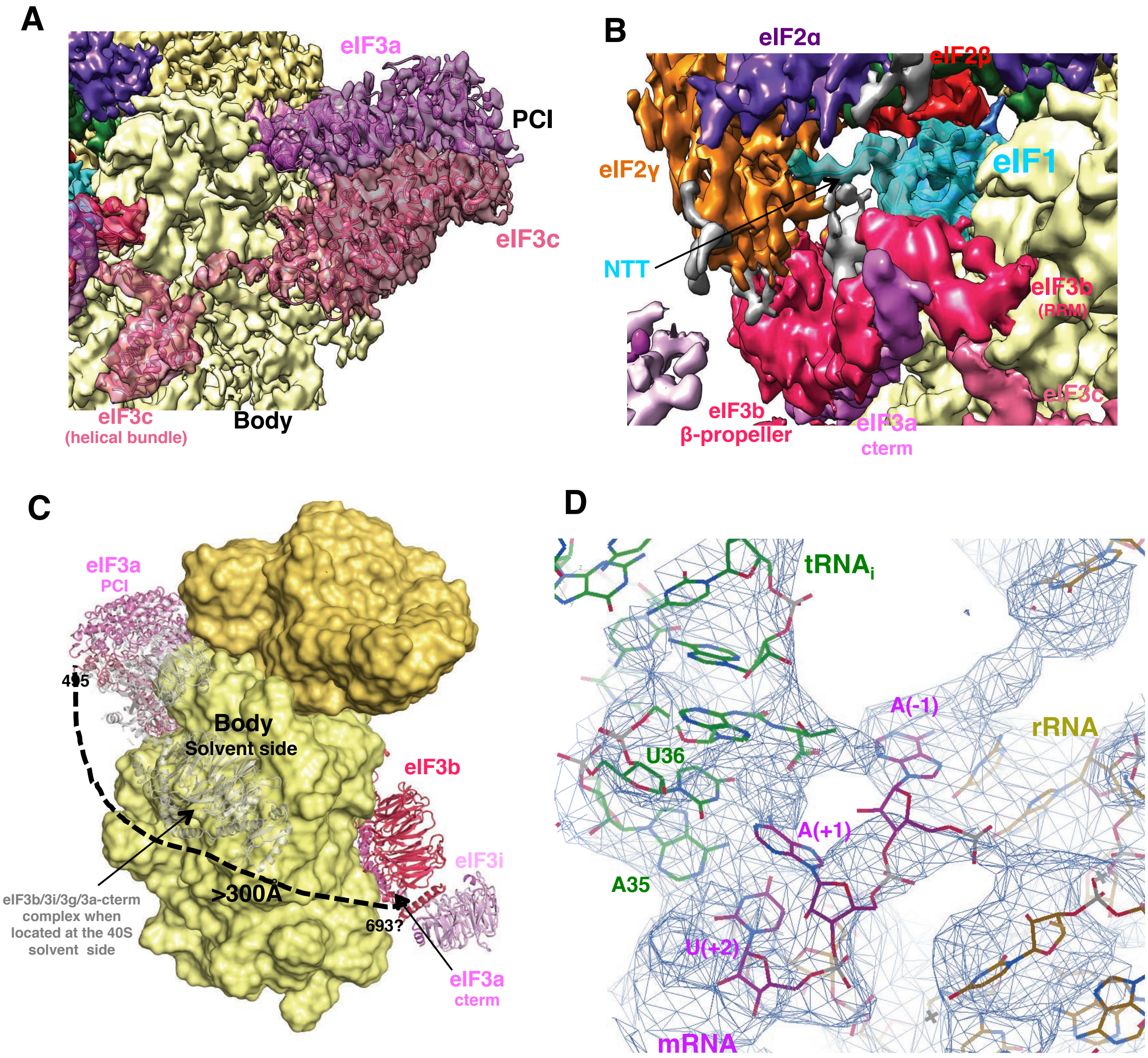

A
eIF3a
PCI
eIF3c
eIF3c
(helical bundle)
Body
B
eIF2α
eIF2β
eIF2γ
eIF1
NTT
eIF3b
(RRM)
eIF3b
β-propeller
eIF3a
cterm
eIF3c
C
eIF3a
PCI
495
Body
Solvent side
eIF3b
eIF3i
>300Å
693?
eIF3b/3i/3g/3a-cterm
complex when
located at the 40S
solvent side
eIF3a
cterm
D
tRNA$_i$
A(-1)
rRNA
U36
A(+1)
A35
U(+2)
mRNA

Figure S6

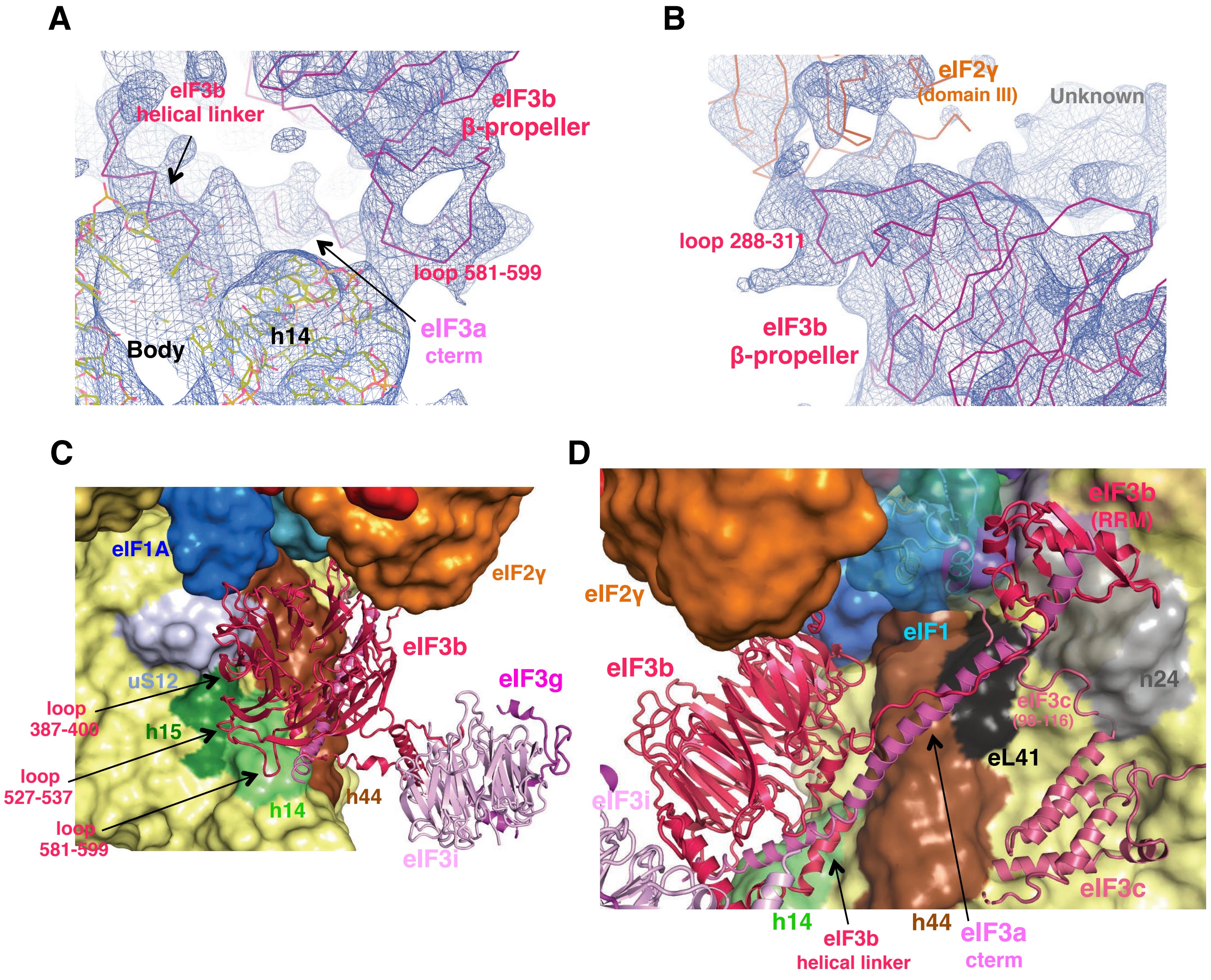
A
eIF3b
helical linker
eIF3b
β-propeller
loop 581-599
eIF3a
cterm
Body
h14
B
eIF2γ
(domain III)
Unknown
loop 288-311
eIF3b
β-propeller
C
eIF1A
eIF2γ
eIF3b
eIF3g
uS12
loop
387-400
h15
loop
527-537
h14
h44
loop
581-599
eIF3i
D
eIF3b
(RRM)
eIF2γ
eIF1
eIF3b
h24
eIF3c
(98-116)
eL41
eIF3i
eIF3c
h14
h44
eIF3b
helical linker
eIF3a
cterm

Figure S7

A

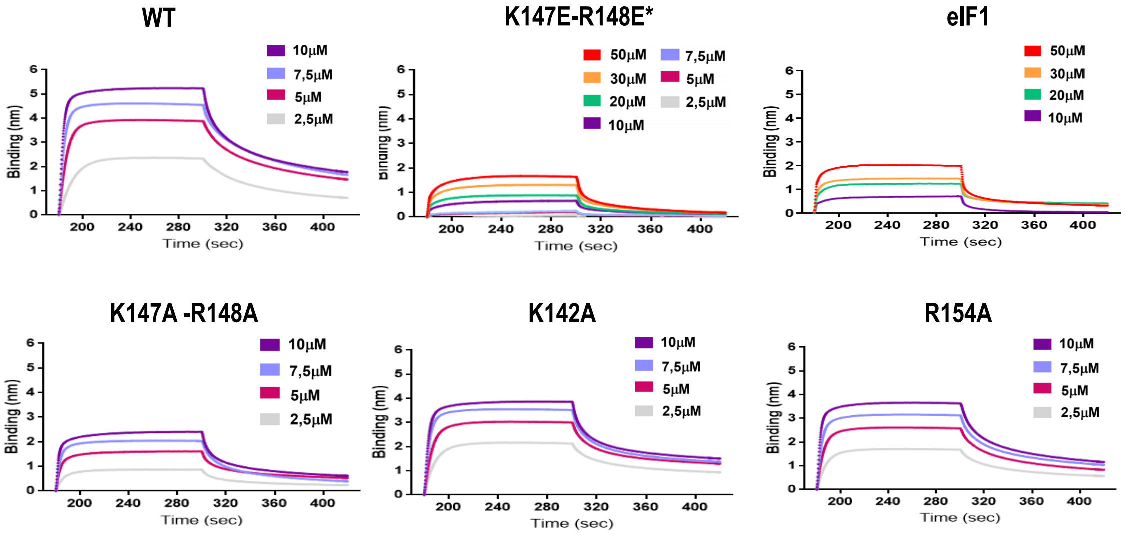

B

	KD	St. dev. KD	Ka	Error Ka	Kd	Error Kd	R^2	p-Value	
WT	2,09E-06	1,32E-06	1,72E+04	9,75E+02	2,56E-02	9,57E-04	0,96	1,00	
K147E-R148E	2,66E-05	1,38E-05	5,79E+03	4,73E+02	6,68E-02	1,88E-03	0,97	0,0314	*
K147A-R148A	4,52E-06	3,82E-06	1,78E+04	8,36E+02	4,46E-02	1,11E-03	0,98	0,2809	
K142A	1,26E-06	3,32E-07	2,24E+04	3,00E+02	1,94E-02	2,39E-04	0,99	0,3816	
R154A	1,13E-06	5,49E-07	2,89E+04	4,13E+02	3,74E-01	7,21E-03	0,98	0,3188	
eIF1	9,33E-06	7,79E-07	6,32E+03	1,27E+03	4,58E-02	1,23E-03	0,96	-	-

Figure S8

py48S-open-eIF3

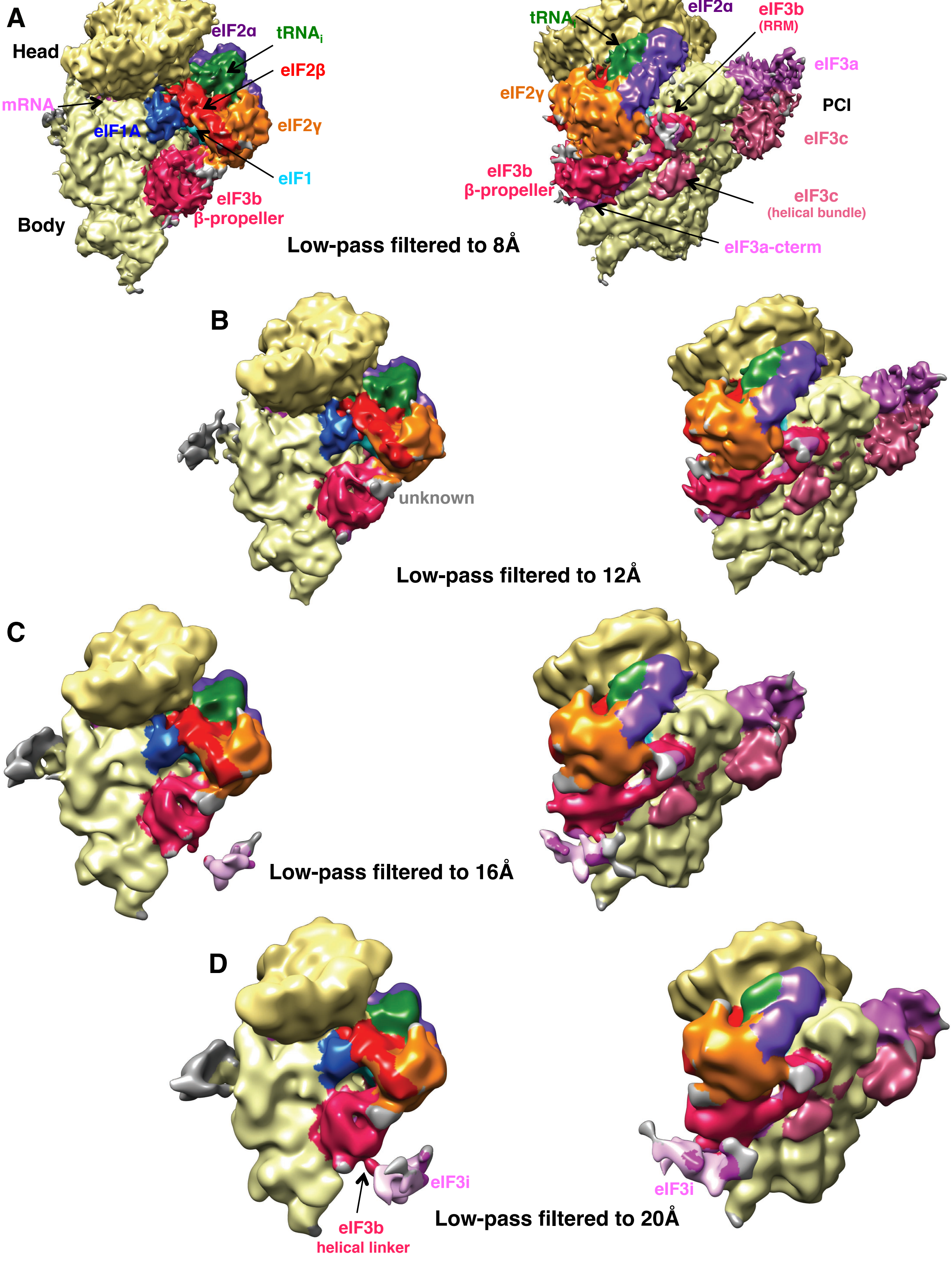

Figure S9

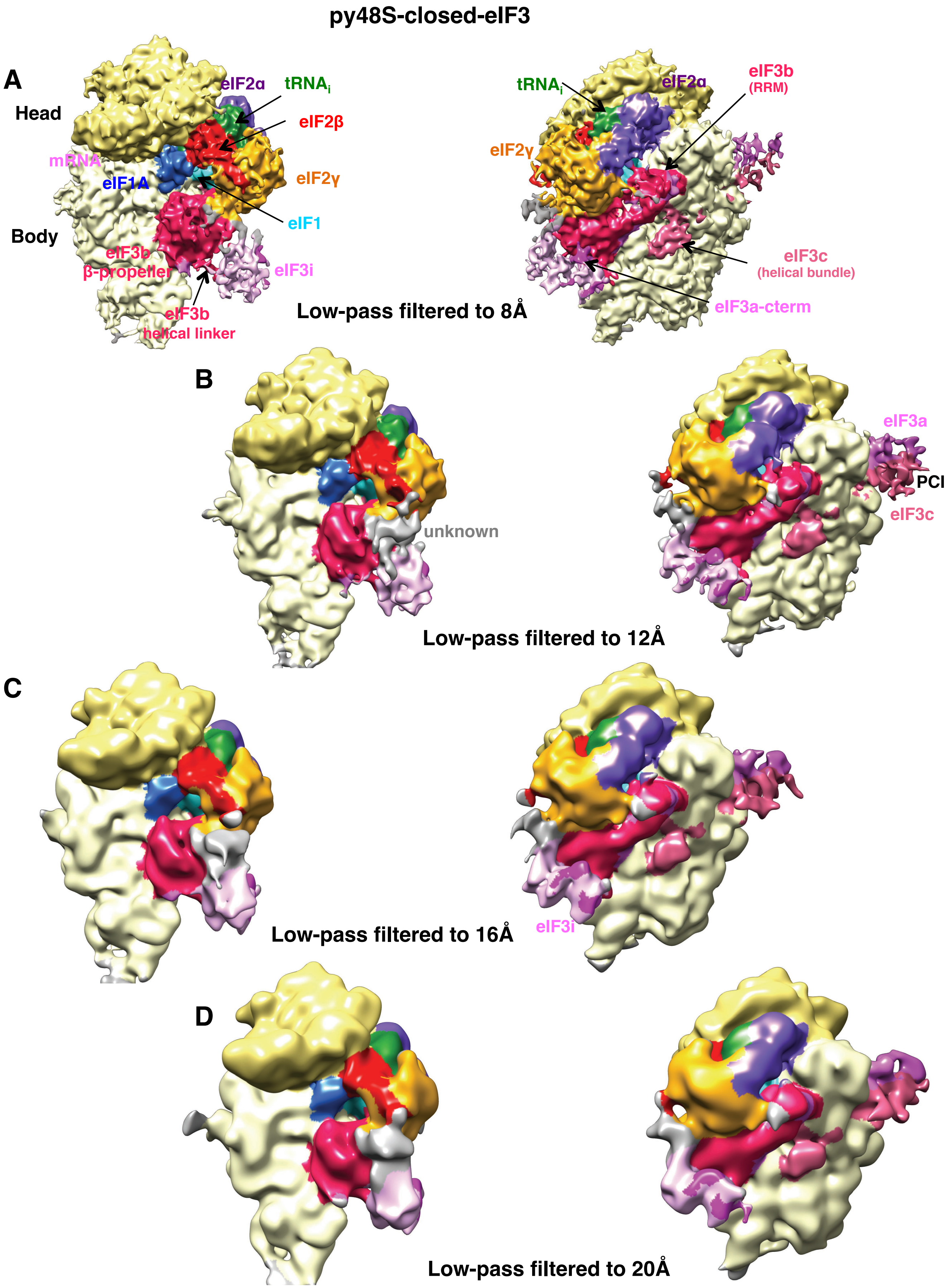
py48S-closed-eIF3
A
Head
Body
mRNA
eIF1A
eIF2α
tRNAi
eIF2β
eIF2γ
eIF1
eIF3b
β-propeller
eIF3i
eIF3b
helical linker
Low-pass filtered to 8Å
tRNAi
eIF2α
eIF3b
(RRM)
eIF2γ
eIF3c
(helical bundle)
eIF3a-cterm
B
unknown
Low-pass filtered to 12Å
eIF3a
PCI
eIF3c
C
Low-pass filtered to 16Å
eIF3i
D
Low-pass filtered to 20Å

Figure S10

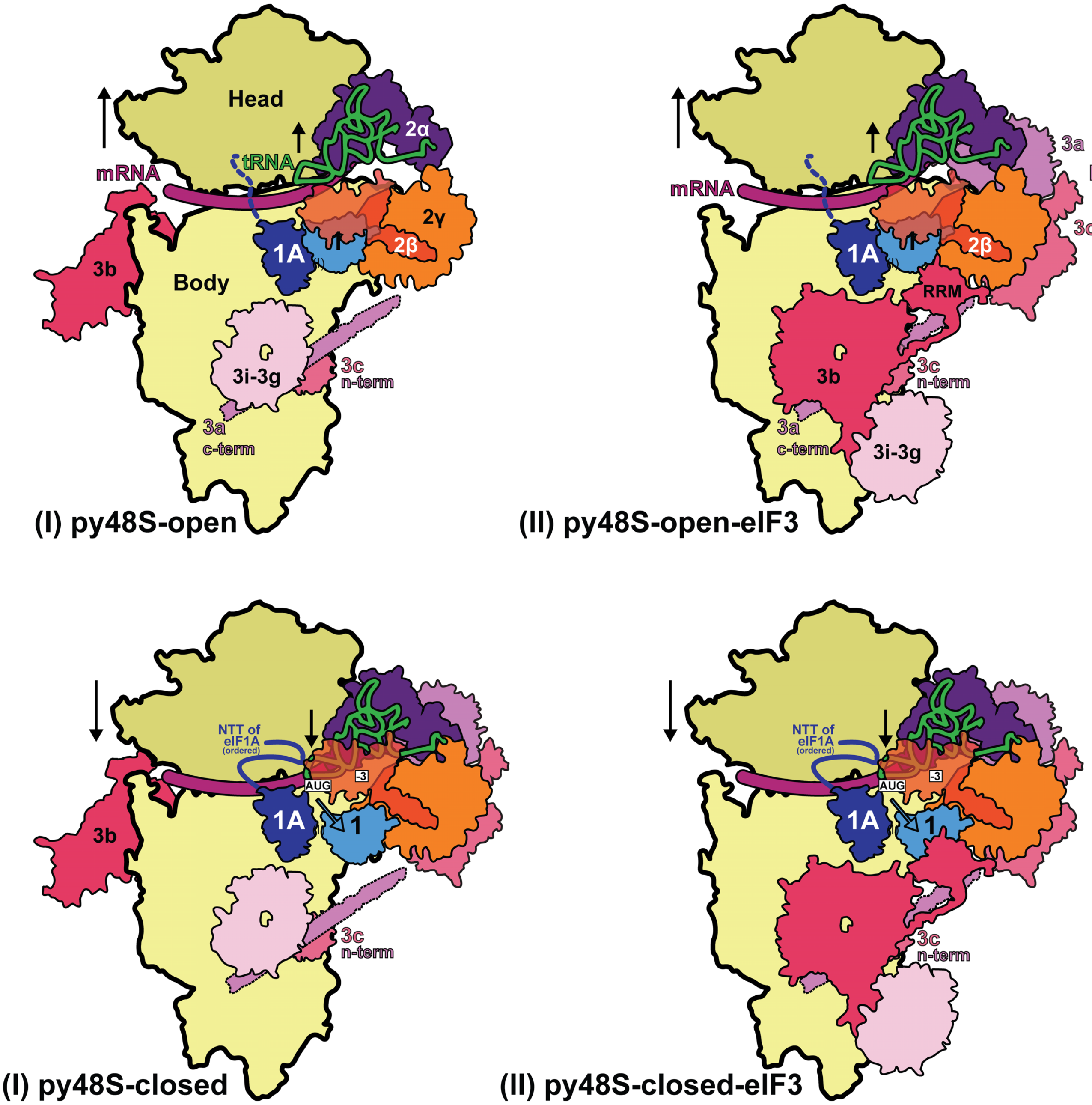
Head
2α
tRNA
mRNA
2γ
2β
1A
3b
Body
3i-3g
3c
n-term
3a
c-term
(I) py48S-open
3a
PCI
3c
RRM
(II) py48S-open-eIF3
NTT of
eIF1A
(ordered)
AUG
-3
1
(I) py48S-closed
(II) py48S-closed-eIF3

A

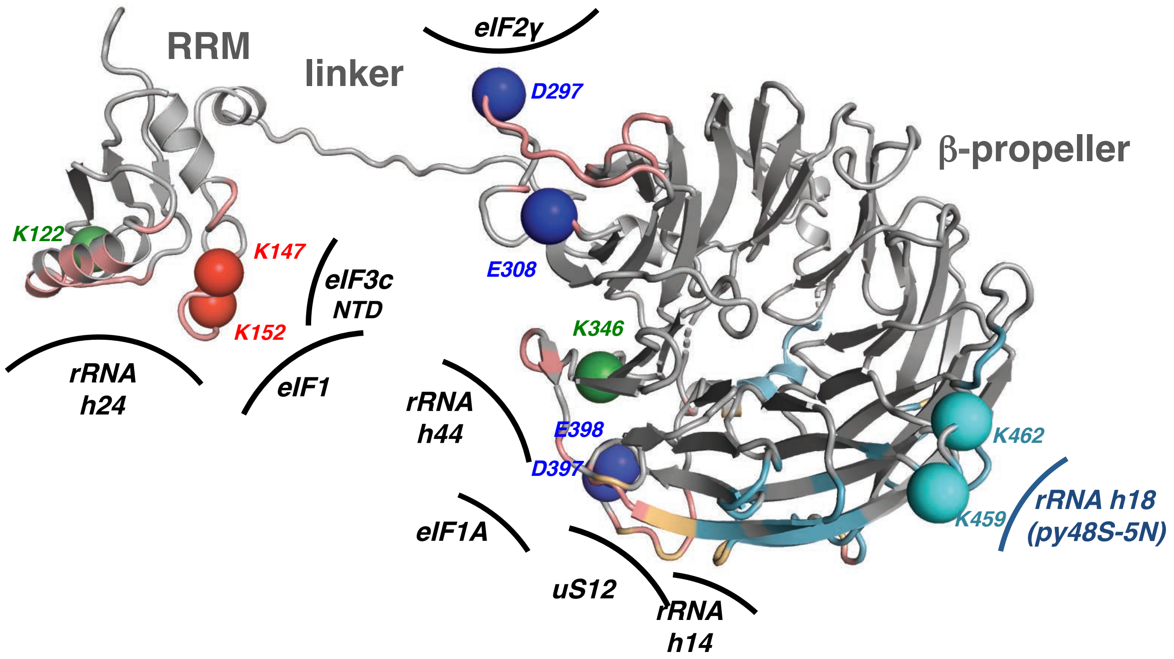

B

MKNFLPRTLKNIYELYFNNISVHSIVSRNTQLKRSKIIQMTTETFEDIKLEDIPVDDIDFSDLEEQYKVT 70

RRM domain

EEFNFDQYIVVNGAPVIPSAKVPVLKKALTSLFSKAGKVVNMEFPIDEATG**K**TKGFLFVECGSMNDAKKI 140

RRM domain

IKSFHG**K**RLDL**K**HRLFLYTMKDVERYNSDDFDTEFREPDMPTFVPSSSLKSWLMDDK*VRDQFVLQDDVKT* 210

SVFWNSMFNEEDSLVESRENWSTNYVRFSPKGTYLFSYHQQGVTAWGGPNFDRLRRFYHPDVRNSSVSPN 280

*EKYLVTFSTEPIIVEE**D**NEFSPFTKKN**E**GHQLCIWDIASGLLMATFPVIKSPYLKWPLVRWSYND**K**YCAR* 350

*MVGDSLIVHDATKNFMPLEAKALKPSGIRDFSFAPEGVKLQPFRNG**DE**PSVLLAYWTPETNNSACTATIA* 420

*EVPRGRVLKTVNLVQVSNVTLHWQNQAEFLCFNVERHT**K**SG**K**TQFSNLQICRLTERDIPVEKVELKDSVF* 490

EFGWEPHGNRFVTISVHEVADMNYAIPANTIRFYAPETKEKTDVIKRWSLVKEIPKTFANTVSWSPAGRF 560

VVVGALVGPNMRRSDLQFYDMDYPGEKNINDNNDVSASLKDVAHPTYSAATNITWDPSGRYVTAWSSSLK 630

*HKVEHGYKIFNIAGNLVKEDIIAGFKNFAWRPR*PASILSNAERKKVRKNLREWSAQFEEQDAMEADTAMR 700

DLILHQRELLKQWTEYREKIGQEMEKSMNFKIFDVQPEDASDDFTTIEEIVEEVLEETKEKVE 763

Figure S12

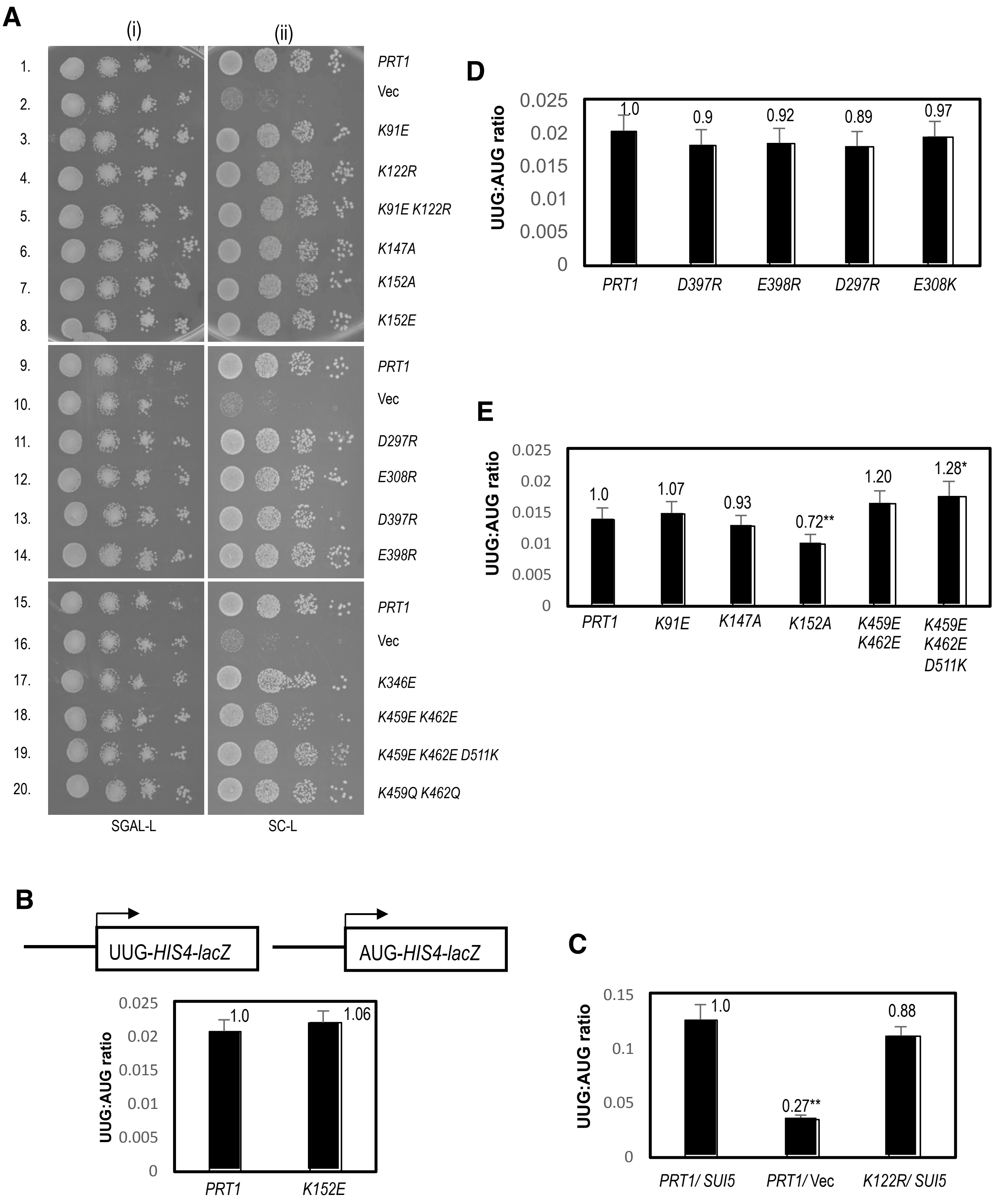

A
(i)
(ii)
1.
2.
3.
4.
5.
6.
7.
8.
9.
10.
11.
12.
13.
14.
15.
16.
17.
18.
19.
20.
PRT1
Vec
K91E
K122R
K91E K122R
K147A
K152A
K152E
PRT1
Vec
D297R
E308R
D397R
E398R
PRT1
Vec
K346E
K459E K462E
K459E K462E D511K
K459Q K462Q
SGAL-L
SC-L
B
UUG-HIS4-lacZ
AUG-HIS4-lacZ
UUG:AUG ratio
0.025
0.02
0.015
0.01
0.005
0
1.0
1.06
PRT1
K152E
C
UUG:AUG ratio
0.15
0.1
0.05
0
1.0
0.27**
0.88
PRT1/ SUI5
PRT1/ Vec
K122R/ SUI5
D
UUG:AUG ratio
0.025
0.02
0.015
0.01
0.005
0
1.0
0.9
0.92
0.89
0.97
PRT1
D397R
E398R
D297R
E308K
E
UUG:AUG ratio
0.025
0.02
0.015
0.01
0.005
0
1.0
1.07
0.93
0.72**
1.20
1.28*
PRT1
K91E
K147A
K152A
K459E K462E
K459E K462E D511K

Figure S13

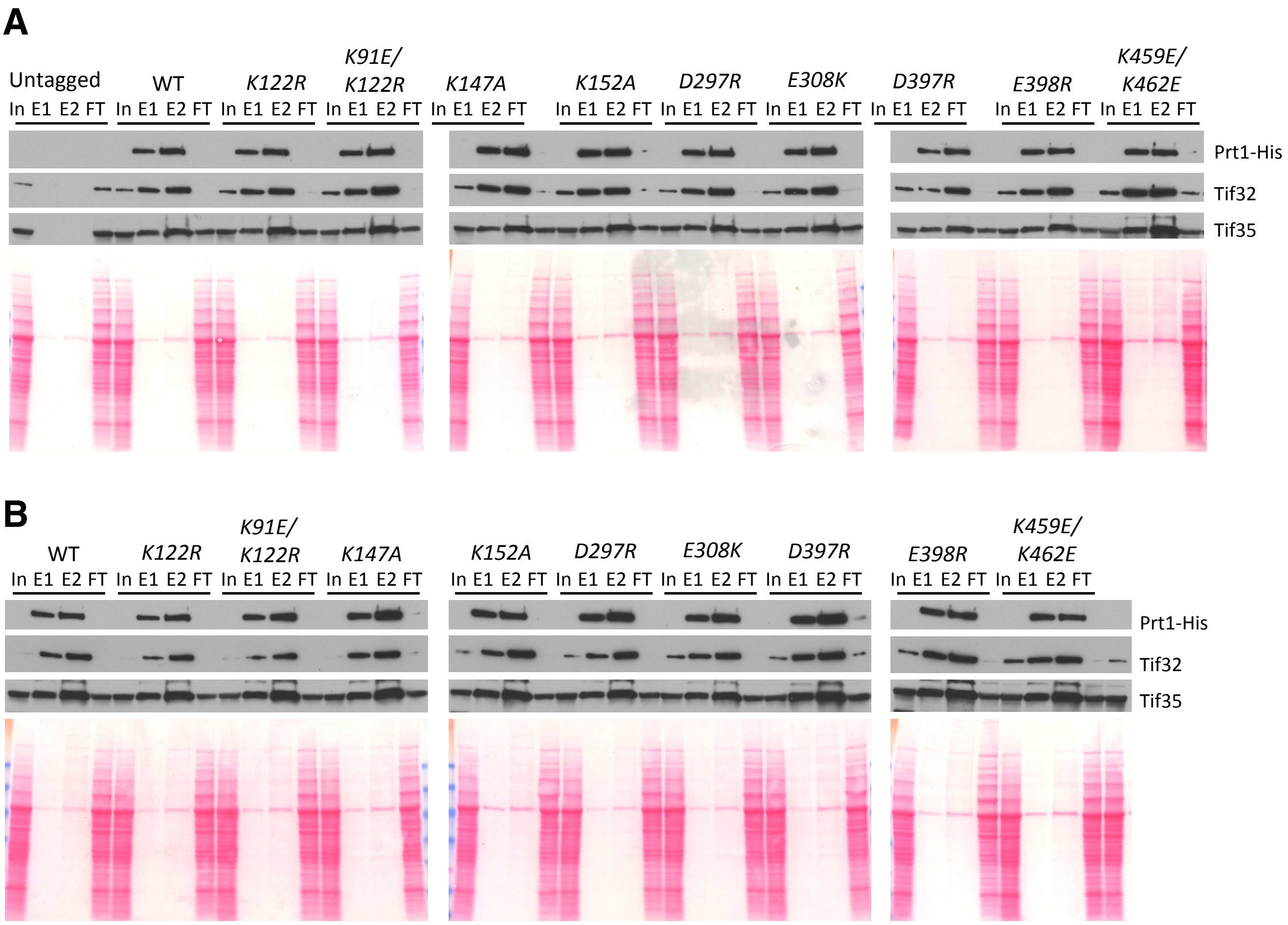
A
Untagged
WT
K122R
K91E/
K122R
K147A
K152A
D297R
E308K
D397R
E398R
K459E/
K462E
In E1 E2 FT
Prt1-His
Tif32
Tif35
B
WT
K122R
K91E/
K122R
K147A
K152A
D297R
E308K
D397R
E398R
K459E/
K462E
In E1 E2 FT
Prt1-His
Tif32
Tif35

Supplementary Figures

Supplementary Figure S1. Cryo-EM methods

(A) Typical micrograph of py48S-open-eIF3 particles (see Material & Methods for details).

(B) Representative 2-D classes after 2-D classification of py48S-open-eIF3 particles.

(C) Gold-standard Fourier Shell Correlation (FSC) curves for the py48S-open-eIF3 and py48S-closed-eIF3 maps.

(D) Scheme of 3D classification of data

For py48S-open-eIF3 (*left*), 360,729 particles were selected after 2D classification and an initial 3D refinement was done. After a 3D-classification into twelve different classes, three classes contained clear density for TC and were grouped together (144,292 particles). Then focused 3D classifications were carried out, using separately a 'TC mask' first (selecting 104,792 particles) and then an 'eIF3 mask' (shown by outlines). As a result, we obtained a class with high occupancy for eIF3 at the subunit interface (13,038 particles) that could be further divided into two classes showing a different degree of head tilting. One of them, dubbed py48S-open-eIF3, corresponds to the fully open head conformation (5,750 particles, at 5.2 Å overall resolution). (*right*) The previous py48S-closed dataset was reprocessed, as follows. 1,182,041 particles were selected after 2D classification and an initial 3D refinement was done. After a 3D-classification into sixteen classes, four classes contained clear density for TC and were joined together. Focused 3D classifications were then carried out. The eIF3 masks 'bgi mask' and 'eIF3 mask' as well as the 'tRNA/alpha mask' used successively for focused 3D classifications are shown in outline. A final class of 12,586 particles showing higher occupancy for eIF3 was obtained, dubbed py48S-closed-eIF3 at 5.8 Å overall resolution. See 'Analysis and structure determination' section of Material & Methods for additional details.

Supplementary Figure S2. Map quality and local resolution

Surface (left or top) and cross-sections (right or bottom) of gaussian-filtered maps, colored according to local resolution.

(A) py48S-open-eIF3, front view

(B) py48S-open-eIF3, lateral view

(C) py48S-closed-eIF3, front view

(D) py48S-closed-eIF3, lateral view

Supplementary Figure S3. Structural features of *S. cerevisiae* eIF3 subunits modeled in the maps

Structures of domains modeled in this study are shown as ribbon diagrams below linear schematics of the primary structures of the corresponding of eIF3 subunits, with locations of the structurally defined domains in the linear schematics shown with hatching and colored as in the ribbon diagrams. Domains/regions of eIF3 not modeled in this study are colored white, and predicted unstructured regions are shown as thinner white rectangles in the linear schematics. Structures not based on previous high-resolution crystal structures, and therefore based on previous low-resolution structures (PDB:5K1H, PDB:3JAP), or modeled "*de novo*" here as polyalanines, are highlighted with an asterisk. Linkers of unknown and un-modeled structure connecting the structured domains of eIF3a or eIF3c are shown as dashed curves in panel A.

(A) Domain map and structures of eIF3a and eIF3c.

(B) Domain map and structure of eIF3b.

(C) Domain map and structures of eIF3i and eIF3g.

Supplementary Figure S4. Proposed model for the N-terminal region of eIF3c and its contacts with other components in py48S-open-eIF3/py48S-closed-eIF3 PICs

(A) Amino acid sequence, secondary structure prediction and sequence conservation of the eIF3c N-terminal region enabled the model building (residues 98 to 226). The β-strands and α-helices are depicted as arrows and rectangles, respectively. Yellow rectangles account for the consensus between the three programs used for secondary structure prediction (see Methods section), whereas those in white correspond to the prediction of at least two of these programs. Residues shown in red are invariant whereas residues with only conservative replacements are in blue in a multiple sequence alignment (see Methods section).

(B,C) Two different views of the eIF3c 5-helical bundle showing the connectivity between the five helices. Residue numbers at the beginning and end of each helix are labeled, exhibiting substantial agreement with the secondary structure predictions in (A).

(D) The side chains of several residues in the eIF3c 5-helical bundle are visible, especially those in contact with rRNA. Two very conserved basic residues (R201, K204) interacting with h11 are labeled.

(E,F) Surface electrostatic representations of 40S proteins, eIF1, and the eIF3b RRM domain and cartoon representations of rRNA and the eIF3c N-terminal region. The C_β for each residue with hydrophobic, basic or acidic sidechains of eIF3c is represented as white, blue and red spheres, respectively. Residue numbers at the beginning and end of each helix are also labeled. In (E), it can be seen that most of the hydrophobic residues are buried, whereas in (E) and (F) the basic residues are exposed and interact mainly with rRNA, whereas the acidic residues interact with

the basic surfaces of eIF1 and eIF3b RRM, as well as with eL41 and uS15. Most of the eIF3c residues involved in these contacts are highly conserved.

(G) Amino acid sequence, secondary structure prediction and sequence conservation of the eIF3a C-terminal region, all depicted as in A.

Supplementary Figure S5. eIF3 domains in py48S-open-eIF3 PIC

(A) Fitting of the eIF3a/c PCI heterodimer in the py48S-open-eIF3 map. Map for eIF3a/c PCI heterodimer is gaussian-filtered by 1.34 and displayed at threshold of 0.025.

(B) Different view of the contacts of the eIF3b β-propeller, eIF3b RRM domain, eIF3a-CTD and most of the N-terminal portion of eIF3c with one another and with different parts of the 40S subunit (colored variously), eIF2γ and eIF1. The N-terminal tail of eIF1 (transparent cyan surface) approaches and makes contacts with eIF2γ.

(C) Proposed path along the solvent side of the 40S subunit for the central portion of eIF3a connecting the eIF3a/PCI domain and eIF3a C-term helix in py48S-closed-eIF3. A linker more than 300 Å long is needed to connect the C-end of the eIF3a PCI domain with the N-end of the eIF3a C-terminal helix that interacts with eIF3b, spanning eIF3a residues 495-693. A semitransparent cartoon representation of the eIF3b-3i-3g-3a-Cter complex is shown at the subunit interface connected to the predicted central eIF3a linker on the solvent-exposed side of the 40S subunit (dashed line), which is presumed to be fully extended to permit re-location of the eIF3b-3i-3g-3a-Cter module to the 40S subunit interface.

(D) Detailed view of the codon-anticodon helix of py48S-open-eIF3. There is only density for the first two bases of the codon, A(+1) and U(+2), and the preceding base A(-1).

Supplementary Figure S6. Fitting and contacts of eIF3b with other components in the py48S-closed-eIF3/py48S-open-eIF3 PICs

(A,B) Fitting of some well-resolved loops of the eIF3b β-propeller into the py48S-closed-eIF3 map.

(C) View of the contacts of the eIF3b β-propeller with different parts of the 40S subunit (colored variously), eIF2γ and the eIF3a-CTD.

(D) View of the contacts of the eIF3b β-propeller, eIF3b RRM domain, eIF3a-CTD and most of the N-terminal portion of eIF3c with one another and with different parts of the 40S subunit (colored variously), eIF2γ and eIF1.

Supplementary Figure S7. Biolayer interferometry assays show the impact of key point mutations in eIF3b RRM on the binding affinity between this domain and eIF3c-NTD

(A) The profiles were obtained using GST-eIF3c-$NTD_{1\text{-}116}$ as bait and increasing concentrations of eIF3b RRM wild type (WT), eIF3b RRM mutants and eIF1.

(B) Table shows the different binding affinities (K_D) and association (ka) and dissociation (kd) constant values for GST-eIF3c-$NTD_{1\text{-}116}$ complex formation with the different eIF3b RRM variants or eIF1. Standard deviation and constant errors are also shown. Values shown are the mean from three experiment replicates. Asterisks indicate significant differences between mutant and WT as judged by a Student's t-test (*$p<0.05$).

Supplementary Figure S8. Low-pass filtered maps of the cryo-EM structure of the py48S-open-eIF3 PIC, shown in two orientations

In addition to the map at 5.2 Å-resolution shown in Figure 1A, a gradual progression of filters to lower resolution is presented to appreciate the presence of density at different resolutions. For example, density for eIF3i and the entire eIF3b helical linker is seen only in the map low-pass filtered to 20 Å in panel D.

(A) Low-pass filtered to 8 Å. Threshold of 0.008. Colored as in Figure 1A.

(B) Low-pass filtered to 12 Å. Threshold of 0.006. Colored as in Figure 1A.

(C) Low-pass filtered to 16 Å. Threshold of 0.004. Colored as in Figure 1A.

(D) Low-pass filtered to 20 Å. Threshold of 0.0025. Colored as in Figure 1A.

Supplementary Figure S9. Low-pass filtered maps of the cryo-EM structure of the py48S-closed-eIF3 PIC, shown in two orientations

In addition to the map at 5.8 Å-resolution shown in Figure 2A, a gradual progression of filters to lower resolution is presented to appreciate the presence of density at different resolutions. For example, density for eIF3i and the entire eIF3b helical linker is already visible in a map low-pass filtered to 8 Å; whereas density for the entire eIF3a/eIF3c PCI domain heterodimer is seen only in the map low-pass filtered to 20 Å. Note that density for the PCI domains of eIF3a and eIF3c was observed earlier at higher resolution for py48S-closed (12).

(A) Low-pass filtered to 8 Å. Threshold of 0.015. Colored as in Figure 2A.

(B) Low-pass filtered to 12 Å. Threshold of 0.003.

(C) Low-pass filtered to 16 Å. Threshold of 0.0025.

(D) Low-pass filtered to 20 Å. Threshold of 0.002.

Supplementary Figure S10. Model showing the differences in modelling between py48S-open-eIF3/ py48S-closed-eIF3 complexes and py48S-open/py48S-closed complexes from our earlier study.

In our earlier study (I), in both py48S-open and py48S-closed: (i) eIF3b was placed on 40S solvent exposed interface, interacting with 40S; (ii) tentatively eIF3i was placed on 40S subunit interface, interacting with 40S; and in the case of py48S-open only, (iii) no density for eIF3a/ eIF3c PCI domains was found.

In this study (II), in both py48S-open-eIF3 and py48S-closed-eIF3: (i) eIF3b is placed on 40S subunit interface; (ii) eIF3i relocates to subunit interface along with eIF3b and does not interact with 40S; (iii) density for eIF3a/ eIF3c PCI domains observed

Supplementary Figure S11. eIF3b contacts and location on the structure of the substituted residues used on the genetics study

(A) Cartoon and transparent surface representation of eIF3b in py48S-open-eIF3. Surfaces colored in salmon encompass residues interacting with the 40S, eIF1, eIF3c or eIF2γ in either py48S-open-eIF3 or py48S-closed-eIF3; surface colored in cyan encompasses residues interacting with the 40S in py48S-5N (PDB: 6FYX); surface colored in yellow includes residues involved in interactions at both the subunit interface and solvent side of the 40S in either py48S-open-eIF3 or py48S-closed-eIF3 and also py48S-5N. Residues substituted in genetic studies are shown as spheres. Based on the phenotypes of the substitutions, red spheres correspond to RRM residues that preferentially stabilize the closed conformation of the py48S-closed-eIF3 PIC, green spheres correspond to residues preferentially stabilizing the open conformation of the py48S-open-eIF3 PIC, blue and cyan spheres correspond to residues facilitating relocation of the

eIF3b/eIF3g/eIF3i module to the 40S solvent-exposed surface either through repulsive interactions with rRNA or eIF2γ at the subunit interface (blue) or attractive interactions with rRNA on the solvent side of the 40S (cyan). Different parts of eIF3b, as well as its interaction partners at the 40S-subunit-interface are labeled. (B) Amino acid sequence of yeast eIF3b/Prt1. Residues substituted in genetic studies are in bold and colored as in (A). Residues in italics belong to the β-propeller of eIF3b. Residues having decreased accessibility [analyzed using a water probe of 1.4A in PISA (53)] upon binding to 40S/eIF1/eIF3c/eIF2γ in py48S-closed-eIF3 (black circles), py48S-open-eIF3 (transparent circles), or to 40S in py48S-5N (PDB: 6FYX; green triangles) are indicated.

Supplementary Figure S12. Supporting phenotypic analysis of *prt1* alleles. (A) Effects of *prt1* alleles on growth in complete medium in otherwise WT cells. Serial dilutions of the P_{GAL}*-PRT1 his4-301* strain HD3607 harboring the indicated plasmid-borne *PRT1* alleles or empty vector (Vec) were spotted on SGAL-L or SC-L medium and incubated for 3-4 d at 30^0 C. **(B, D, E) Effects of selected *prt1* alleles on the *HIS4-lacZ* UUG:AUG initiation ratio in otherwise WT cells.** Transformants of P_{GAL1}*-PRT1 his4-301* strain HD3607 with the indicated *PRT1* alleles and *HIS4-lacZ* fusions with AUG or UUG start codons were cultured in SD+His+Trp, and analyzed exactly as in Figure 5B. **(C) *prt1-K112R* does not suppress the increased *HIS4-lacZ* UUG:AUG initiation ratio conferred by *SUI5***. The P_{GAL}*-PRT1 his4-301* strain HD3607 with the indicated plasmid-borne *PRT1* allele and harboring a single copy (sc) *SUI5* plasmid or empty vector (Vec), and *HIS4-lacZ* fusions with AUG or UUG start codons were analyzed exactly as in Figure 5B.

Supplementary Figure S13. Transformants of HD3607 harboring the indicated WT or mutant alleles of *PRT1-His* on *LEU2* plasmids (described in Supplementary Figure S11A), or an HD3607 transformant harboring low-copy *URA3* plasmid p2625 containing WT *PRT1* lacking the His_8 tag (Untagged), were cultured in SC-Leu medium (or SC-Ura for the Untagged control strain) to A_{260} of ca. 1.0. Whole cell extracts (WCEs) were incubated with Ni-NTA-silica resin, bound proteins were eluted and 5% of the input WCE (In), 30% (E1) or 60% (E2) of the eluate, and 5% of the flow-through for each sample were subjected to Western blot analysis with antibodies against the His_8 epitope (for Prt1-His proteins; abCam cat. # ab18184), eIF3a/Tif32, or eIF3g/Tif35 (upper panels). Ponceau S staining of the blots is shown below (lower panels). Immune complexes were visualized using enhanced chemiluminescence reagent (Amersham). The results obtained for two biological replicates are shown in panels A and B, respectively.

Movie 1. Movie showing the transition from py48S-open-eIF3 to py48S-closed-eIF3 PICs, in two different orientations and highlighting the subtle rearrangement of eIF3 elements at the subunit interface in this rearrangement.

Movie 2. Movie showing the internal rearrangement in the eIF3b/eIF3i/eIF3g/eIF3a-Cterm quaternary complex found on the 40S solvent side in py48S-eIF5N (PDB: 6FYY) and that on py48S-closed-eIF3 PICs on the 40S subunit interface. eIF1 is also shown, in cyan.

Movie 3. Movie depicting the structural transitions in 43S/48S PICs summarized in the cartoon of Figure 7. It shows the two different locations observed for the eIF3b/eIF3i/eIF3g/eIF3a-Cterm module on the solvent surface of the 40S subunit in p43S and py48S-eIF5N, and on the subunit interface in the py48S-open-eIF3/py48S-closed-eIF3 PICs. The movie begins with the p43S PIC,

where the eIF3a/3c PCI domains and eIF3b/eIF3i/eIF3g/eIF3a-Cterm module are all at the solvent interface. In py48S-open-eIF3, the 40S head moves up with respect to the body and the eIF3b/eIF3i/eIF3g/eIF3a-Cterm module relocates to the subunit interface. Next, upon start codon recognition in py48S-closed-eIF3 PIC, the head moves down in transition to a closed conformation of the 40S while the eIF3b/eIF3i/eIF3g/eIF3a-Cterm module undergoes a subtle repositioning on the subunit interface. eIF1 is present in py48S-closed-eIF3 PIC. Thereafter, eIF1 dissociates from the PIC, eIF5-NTD binds at the P site and occupies the position left vacant by eIF1, and the eIF3b/eIF3i/eIF3g/eIF3a-Cterm module relocates back to the solvent interface in py48S-eIF5N PIC.

Table S1. Summary of phenotypes of *PRT1* alleles

PRT1 allele	Cell Growth at on SC	[1]Sui$^-$	Suppression of *SUI5* His$^+$	Suppression of *SUI5* Slg$^-$ at 37°C	*HIS4-lacZ* UUG:AUG in *SUI5*	Suppression of *SUI5* UUG:AUG
WT	++++	none	None	none	0.15±0.012	none
K91E	++++	none	None	none	NA	NA
K122R	++++	Weak	None	none	0.13±0.008	none
K91E K122R	++++	none	None	none	ND	ND
K147A	++++	none	Strong	Strong	0.036±0.001	Strong
K152E	++++	none	Strong	Strong	0.033±0.005	Strong
K152A	++++	none	Modest	strong	0.082±0.009	Modest
D397R	++++	none	Strong	Strong	0.042±0.004	Strong
E398R	++++	none	Strong	Strong	0.050±0.006	Strong
D297R	++++	none	None	Strong	0.014±0.001	Strong
E308K	++++	none	None	Strong	0.006±0.002	Strong
K346E	++++	Weak	none*	none*	ND	ND
K459E K462E	++	none	Strong	Strong$^-$	0.039±0.005	Strong
K459Q K462Q	++++	ND	Strong	Strong	0.075±0.010	Modest
K459E K462E D511K	+++	none	None	none	0.15±0.009	none

[1]as judged by *HIS4-lacZ* UUG:AUG initiation ratio in strains lacking *SUI5:* none indicates an essentially WT ratio; weak indicates an ≈2-fold increase compared to the WT ratio.

NA: Not Applicable. Extreme Slg$^-$ in the presence of *SUI5* prevented introduction of *HIS4-lacZ* reporter plasmids.
ND: Not Determined
*Data not shown in figures

Table S2. Primers used for mutagenesis (with mutated positions underlined)

Name	Sequence	Mutation
K91E	5' GTCATCCCATCCGCCGAAGTTCCTGTTTTGAAAAAG	*K91E*
K91E-r	5' CTTTTTCAAAACAGGAACTTCGGCGGATGGGATGAC	*K91E*
K122R	5' GATGAAGCCACTGGTAGGACGAAAGGTTTTCTCTTCG	*K122R*
K122R-r	5' CGAAGAGAAAACCTTTCGTCCTACCAGTGGCTTCATC	*K122R*
K147A	5' CAAGAGTTTCCACGGTGAAAGACTGGATTTAAAACATCG	*K147A*
K147A-r	5' CGATGTTTTAAATCCAGTCTTGCACCGTGGAAACTCTTG	*K147A*
K152A	5' GTAAAAGACTGGATTTAGCACATCGTTTGTTTCTTTATAC	*K152A*
K152A-r	5' GTATAAAGAAACAAACGATGTGCTAAATCCAGTCTTTTAC	*K152A*
K152E	5' GTAAAAGACTGGATTTAGAACATCGTTTGTTTCTTTATAC	*K152E*
K152E-r	5' GTATAAAGAAACAAACGATGTTCTAAATCCAGTCTTTTAC	*K152E*
D297R	5' CCAATCATTGTAGAAGAACGTAACGAATTCTCTCCATTTACC	*D297R*
D297R-r	5' GGTAAATGGAGAGAATTCGTTACGTTCTTCTACAATGATTGG	*D297R*
E308K	5' CCATTTACCAAGAAAAATAAGGGTCATCAATGCATC	*E308K*
E308K-r	5' GATGCATTGATGACCCTTATTTTTCTTGGTAAATGG	*E308K*
K346E	5' GATGGTCTTATAATGATGAATATTGTGCTCGTATGGTTGG	*K346E*
K346E-r	5' CCAACCATACGAGCACAATATTCATCATTATAAGACCATC	*K346E*
D397R	5' CAACCATTCAGAAACGGTCGCGAGCCTTCTGTTTTATTG	*D397R*
D397R-r	5' CAATAAAACAGAAGGCTCGCGACCGTTTCTGAATGGTTG	*D397R*
E398R	5' CCATTCAGAAACGGTGACAGGCCTTCTGTTTTATTGGC	*E398R*
E398R-r	5' GCCAATAAAACAGAAGGCCTGTCACCGTTTCTGAATGG	*E398R*
K459E	5' CAATGTCGAACGTCACACAGAATCTGGTAAGACTCAATTC	*K459E*
K459E-r	5' GAATTGAGTCTTACCAGATTCTGTGTGACGTTCGACATTG	*K459E*
K459Q	5' CAATGTCGAACGTCACACACAATCTGGTAAGACTCAATTC	*K459Q*
K459Q-r	5' GAATTGAGTCTTACCAGATTGTGTGTGACGTTCGACATTG	*K459Q*
K462E	5' CGTCACACAAAGTCTGGTGAAACTCAATTCAGTAATCTAC	*K462E*
K462E-r	5' GTAGATTACTGAATTGAGTTTCACCAGACTTTGTGTGACG	*K462E*
K462Q	5' CGTCACACAAAGTCTGGTCAAACTCAATTCAGTAATCTAC	*K462Q*
K462Q-r	5' GTAGATTACTGAATTGAGTTTGACCAGACTTTGTGTGACG	*K462Q*
D511K	5' CTGTTCATGAAGTAGCTAAAATGAATTATGCTATCCCAGC	*D511K*
D511K-r	5' GCTGGGATAGCATAATTCATTTTAGCTACTTCATGAACAG	*D511K*

Table S3. Plasmids employed in this work

Plasmid	Description	Source
pRS315	lc[a] *LEU2* vector	(8)
pRS316	lc *URA3* vector	(8)
p4281	sc[b] *TRP1 TIF5-G31R* (*SUI5*) in YCplac22	(9)
YCplac22	sc *TRP1* vector	(10)
p5188	lc *LEU2, PRT1*$^{+}$ in pRS315	(11)
pDH14-29	lc *LEU2, prt1-K91E*	This study
pDH14-90	lc *LEU2, prt1-K122R*	This study
pDH15-26	lc *LEU2, prt1-K91E K122R*	This study
pDH15-67	lc *LEU2, prt1-K147A*	This study
pDH14-95	lc *LEU2, prt1-K152A*	This study
pDH14-96	lc *LEU2, prt1-K152E*	This study
pDH14-56	lc *LEU2, prt1-D297R*	This study
pDH14-57	lc *LEU2, prt1-E308K*	This study
pDH15-9	lc *LEU2, prt1-D397R*	This study
pDH15-10	lc *LEU2, prt1-E398R*	This study
pDH14-61	lc *LEU2, prt1-K346E*	This study
pDH15-11	lc *LEU2, prt1-K459E K462E*	This study
pDH15-76	lc *LEU2, prt1-K459Q K462Q*	This study
pDH15-42	lc *LEU2, prt1-K459E K462E D511K*	This study
pDH15-59	lc *TRP1, PRT1*$^{+}$	This study
pDH15-61	lc *TRP1, prt1-K122R*	This study
pLT11	Marker Swap plasmid (*LEU2::TRP1* Converter)	(12)
p3218	pFA6a-*kanMX6*-P_{GAL1}	(13)
p367	sc *URA3 HIS4(ATG)-lacZ*	(14)
p391	sc *URA3 HIS4(TTG)-lacZ*	(14)
p4836	*sc LEU2 SUI1*	(15)
p5372	*sc LEU2 sui1-K60E*	(16)

[a]lc, low copy number; [b]sc, single copy.

Table S4. Yeast strains employed in this work.

Strain	Genotype	Source
H2995	*MATa ura3-52 trp1-63 leu2-3, 112 his4-301(ACG)*	(9)
HD3607	*MATa ura3-52 trp1-63 leu2-3, 112 his4-301(ACG) P_{GAL}-PRT1::KanMX*	This study
HD3648	*MATa ura3-52 trp1-63 leu2-3, 112 his4-301(ACG) P_{GAL}-PRT1::KanMX* p5188 [lc *LEU2 PRT1* in pRS315]	This study
HD3687	*MATa ura3-52 trp1-63 leu2-3, 112 his4-301(ACG) P_{GAL}-PRT1::KanMX* pRS315 [lc *LEU2*]	This study
HD3649	*MATa ura3-52 trp1-63 leu2-3, 112 his4-301(ACG) P_{GAL}-PRT1::KanMX* pDH14-29 [lc *LEU2 prt1-K91E* in pRS315]	This study
HD3848	*MATa ura3-52 trp1-63 leu2-3, 112 his4-301(ACG) P_{GAL}-PRT1::KanMX* pDH14-90 [lc *LEU2 prt1-K122R* in pRS315]	This study
HD4059	*MATa ura3-52 trp1-63 leu2-3, 112 his4-301(ACG) P_{GAL}-PRT1::KanMX* pDH15-26 [lc *LEU2 prt1-K91E K122R* in pRS315]	This study
HD4262	*MATa ura3-52 trp1-63 leu2-3, 112 his4-301(ACG) P_{GAL}-PRT1::KanMX* pDH15-67 [lc *LEU2 prt1-K147A* in pRS315]	This study
HD4256	*MATa ura3-52 trp1-63 leu2-3, 112 his4-301(ACG) P_{GAL}-PRT1::KanMX* pDH14-95 [lc *LEU2 prt1-K152A* in pRS315]	This study
HD3850	*MATa ura3-52 trp1-63 leu2-3, 112 his4-301(ACG) P_{GAL}-PRT1::KanMX* pDH14-96 [lc *LEU2 prt1-K152E* in pRS315]	This study
HD3668	*MATa ura3-52 trp1-63 leu2-3, 112 his4-301(ACG) P_{GAL}-PRT1::KanMX* pDH14-56 [lc *LEU2 prt1-D297R* in pRS315]	This study
HD3669	*MATa ura3-52 trp1-63 leu2-3, 112 his4-301(ACG) P_{GAL}-PRT1::KanMX* pDH14-57 [lc *LEU2 prt1-E308K* in pRS315]	This study
HD3861	*MATa ura3-52 trp1-63 leu2-3, 112 his4-301(ACG) P_{GAL}-PRT1::KanMX* pDH15-9 [lc *LEU2 prt1-D397R* in pRS315]	This study
HD3862	*MATa ura3-52 trp1-63 leu2-3, 112 his4-301(ACG) P_{GAL}-PRT1::KanMX* pDH15-10 [lc *LEU2 prt1-E398R* in pRS315]	This study
HD3672	*MATa ura3-52 trp1-63 leu2-3, 112 his4-301(ACG) P_{GAL}-PRT1::KanMX* pDH14-61 [lc *LEU2 prt1-K346E* in pRS315]	This study
HD3955	*MATa ura3-52 trp1-63 leu2-3, 112 his4-301(ACG) P_{GAL}-PRT1::KanMX* pDH15-11 [lc *LEU2 prt1-K459E K462E* in pRS315]	This study

Strain	Genotype	Source
HD4263	*MATa ura3-52 trp1-63 leu2-3, 112 his4-301(ACG)* P_{GAL}*-PRT1::KanMX* pDH15-76 [lc *LEU2 prt1-K459Q K462Q* in pRS315]	This study
HD4075	*MATa ura3-52 trp1-63 leu2-3, 112 his4-301(ACG)* P_{GAL}*-PRT1::KanMX* pDH15-42 [lc *LEU2 prt1-K459E K462E D511K* in pRS315]	This study
HD4081	*MATa ura3-52 trp1-63 leu2-3, 112 his4-301(ACG)* P_{GAL}*-PRT1::KanMX* p5188 [lc *LEU2* $PRT1^{+}$ in pRS315] p4281 [sc *TRP1 SUI5*]	This study
HD4082	*MATa ura3-52 trp1-63 leu2-3, 112 his4-301(ACG)* P_{GAL}*-PRT1::KanMX* p5188 [lc *LEU2* $PRT1^{+}$ in pRS315] Ycplac22 [sc *TRP1*]	This study
HD4269	*MATa ura3-52 trp1-63 leu2-3, 112 his4-301(ACG)* P_{GAL}*-PRT1::KanMX* pDH15-67 [lc *LEU2 prt1-K147A* in pRS315] p4281[sc *TRP1 SUI5*]	This study
HD3995	*MATa ura3-52 trp1-63 leu2-3, 112 his4-301(ACG)* P_{GAL}*-PRT1::KanMX* pDH14-96 [lc *LEU2 prt1-K152E* in pRS315] p4281 [sc *TRP1 SUI5*]	This study
HD4257	*MATa ura3-52 trp1-63 leu2-3, 112 his4-301(ACG)* P_{GAL}*-PRT1::KanMX* pDH14-95 [lc *LEU2 prt1-K152A* in pRS315] p4281 [sc *TRP1 SUI5*]	This study
HD4280	*MATa ura3-52 trp1-63 leu2-3, 112 his4-301(ACG)* P_{GAL}*-PRT1::KanMX* pDH15-9 [lc *LEU2 prt1-D397R* in pRS315] p4281 [sc *TRP1 SUI5*]	This study
HD4281	*MATa ura3-52 trp1-63 leu2-3, 112 his4-301(ACG)* P_{GAL}*-PRT1::KanMX* pDH15-10 [lc *LEU2 prt1-E398R* in pRS315] p4281 [sc *TRP1 SUI5*]	This study
HD3749	*MATa ura3-52 trp1-63 leu2-3, 112 his4-301(ACG)* P_{GAL}*-PRT1::KanMX* pDH14-56 [lc *LEU2 prt1-D297R* in pRS315] p4281 [sc *TRP1 SUI5*]	This study
HD4279	*MATa ura3-52 trp1-63 leu2-3, 112 his4-301(ACG)* P_{GAL}*-PRT1::KanMX* pDH14-57 [lc *LEU2 prt1-E308K* in pRS315] p4281 [sc *TRP1 SUI5*]	This study
HD4012	*MATa ura3-52 trp1-63 leu2-3, 112 his4-301(ACG)* P_{GAL}*-PRT1::KanMX* pDH15-11 [lc *LEU2 prt1-K459E K462E* in pRS315] p4281 [sc *TRP1 SUI5*]	This study
HD4268	*MATa ura3-52 trp1-63 leu2-3, 112 his4-301(ACG)* P_{GAL}*-PRT1::KanMX* pDH15-76 [lc *LEU2 prt1-K459Q K462Q* in pRS315] p4281 [sc *TRP1 SUI5*]	This study
HD4009	*MATa ura3-52 trp1-63 leu2-3, 112 his4-301(ACG)* P_{GAL}*-PRT1::KanMX* pDH15-42[lc *LEU2 prt1-K459E K462E D511K* in pRS315] p4281 [sc *TRP1 SUI5*]	This study
HD3993	*MATa ura3-52 trp1-63 leu2-3, 112 his4-301(ACG)* P_{GAL}*-PRT1::KanMX* pDH14-90 [lc *LEU2 prt1-K122R* in pRS315] p4281 [sc *TRP1 SUI5*]	This study

Strain	Genotype	Source
H3956	*MATa ura3-52 leu2-3,112 trp1-Δ63 his4-303(AUU) sui1Δ::hisG* p1200 [*SUI1, URA3 CEN4*]	This study
HD4053	*MATa ura3-52 leu2-3,112 trp1-Δ63 his4-303(AUU)* P_{GAL}*-PRT1::KanMX sui1Δ::hisG* p1200 [sc *SUI1, URA3 CEN4*].	This study
HD4108	*MATa ura3-52 leu2-3,112 trp1-Δ63 his4-303(AUU)* P_{GAL}*-PRT1 sui1Δ::hisG* p4836 [sc *SUI1, LEU2 CEN4*].	This study
HD4109	*(MATa ura3-52 leu2-3,112 trp1-Δ63 his4-303(AUU)* P_{GAL}*-PRT1::KanMX sui1Δ::hisG* p5372 [sc *sui1-K60E, LEU2 CEN4*].	This study
HD4192	*MATa ura3-52 leu2-3,112 trp1-Δ63 his4-303(AUU)* P_{GAL}*-PRT1::KanMX sui1Δ::hisG p4836* [*SUI1, LEU2 CEN4*] pDH15-59 [lc *PRT1 TRP1*].	This study
HD4193	*MATa ura3-52 leu2-3,112 trp1-Δ63 his4-303(AUU)* P_{GAL}*-PRT1::KanMX sui1Δ::hisG p5372* [*sui1-K60E, LEU2 CEN4*] pDH15-59 [lc *PRT1 TRP1*]	This study
HD4196	*MATa ura3-52 leu2-3,112 trp1-Δ63 his4-303(AUU)* P_{GAL}*-PRT1::KanMX sui1Δ::hisG p4836* [*SUI1, LEU2 CEN4*] pDH15-61 [lc *prt1-K122R TRP1*]	This study
HD4197	*MATa ura3-52 leu2-3,112 trp1-Δ63 his4-303(AUU)* P_{GAL}*-PRT1::KanMX sui1Δ::hisG p5372* [*sui1-K60E, LEU2 CEN4*] pDH15-61[lc *prt1-K122R TRP1*]	This study
